# An AAV Vector-Mediated Gene Delivery Approach Facilitates Reconstitution of Functional Human CD8^+^ T Cells in Mice

**DOI:** 10.1371/journal.pone.0088205

**Published:** 2014-02-06

**Authors:** Jing Huang, Xiangming Li, Jordana G. A. Coelho-dos-Reis, James M. Wilson, Moriya Tsuji

**Affiliations:** 1 HIV and Malaria Vaccine Program, Aaron Diamond AIDS Research Center, Affiliate of The Rockefeller University, New York, New York, United States of America; 2 Gene Therapy Program, Department of Pathology and Laboratory Medicine, University of Pennsylvania, Philadelphia, Pennsylvania, United States of America; University of Kansas Medical Center, United States of America

## Abstract

In the present study, a novel adeno-associated virus (AAV) vector-mediated gene delivery approach was taken to improve the reconstitution of functional CD8^+^ T cells in humanized mice, thereby mimicking the human immune system (HIS). Human genes encoding HLA-A2 and selected human cytokines (A2/hucytokines) were introduced to an immune-deficient mouse model [NOD/SCID/IL2rγ^null^ (NSG) mice] using AAV serotype 9 (AAV9) vectors, followed by transplantation of human hematopoietic stem cells. NSG mice transduced with AAV9 encoding A2/hucytokines resulted in higher levels of reconstitution of human CD45^+^ cells compared to NSG mice transduced with AAV9 encoding HLA-A2 alone or HLA-A2-transgenic NSG mice. Furthermore, this group of HIS mice also mounted the highest level of antigen-specific A2-restricted human CD8^+^ T-cell response upon vaccination with recombinant adenoviruses expressing human malaria and HIV antigens. Finally, the human CD8^+^ T-cell response induced in human malaria vaccine-immunized HIS mice was shown to be functional by displaying cytotoxic activity against hepatocytes that express the human malaria antigen in the context of A2 molecules. Taken together, our data show that AAV vector-mediated gene delivery is a simple and efficient method to transfer multiple human genes to immune-deficient mice, thus facilitating successful reconstitution of HIS in mice. The HIS mice generated in this study should ultimately allow us to swiftly evaluate the T-cell immunogenicity of various human vaccine candidates in a pre-clinical setting.

## Introduction

Small animal models have widely been employed in medical research and drug/vaccine development. However, some important human pathogens, including human immunodeficiency virus (HIV) and dengue virus, display tropism unique to humans. In addition, in the host, the protective immune responses between human and non-human species show significant discrepancy. Due to ethical constraints and the high cost of human clinical trials, it is necessary to search for an alternative animal model that can mimic humans, particularly the human immune system (HIS). In fact, various attempts have been made to establish humanized mice that can mount a human immune response, termed HIS mice [1]–[5]. HIS mice have been generated by engrafting human CD34^+^ hematopoietic stem cells (HSCs) derived from various sources [6] to highly immune-deficient mice, such as NOD/SCID/IL2rγ^null^ (NSG) [7] or NOD/SCID/IL2rγc^null^ (NOG) mice [8]. Because these highly immune-deficient mice lack almost the entire mouse-derived immune system, including murine B cells, T cells, and natural killer cells [7], [8], HSCs can be engrafted without encountering xenograft rejection [7]–[9]. However, for HSCs to develop into human CD45^+^ cells and become fully functional human lymphocytes in mice, human-specific growth factors and cytokines (such as GM-CSF, IL-3, and IL-15) are required [10], [11]. In addition, expression of human-specific major histocompatibility complex (MHC) [i.e., human leukocyte antigens (HLAs)], particularly in the thymus, is essential for human T cells to be properly educated and for their successful development and differentiation [4].

Currently, there are a few ways to facilitate the development of HIS from HSCs engrafted in immune-deficient mice. The first approach is to implant human thymus under the kidney capsule, along with human fetal liver and bone marrow. These are called BLT (bone marrow-liver-thymus) mice [12], [13]. Among the several types of humanized mice developed, the BLT mouse model is by far the most complete and well explored humanized mouse model available to date. This model provides high levels of human stem cell engraftment, allowing development of human myeloid and lymphoid lineages as well as high functional T-cell responses [12]–[22]. However, establishment of the BLT mouse model requires the availability of bone marrow, liver, and thymus from the same human donor, which must be transplanted into mice surgically. The high costs and technical demands of this system considerably increase the research cost, and are sometimes comparable to the expense of studies using non-human primates. Finally, because each mouse needs to be surgically implanted with human thymus, there is always significant variation among BLT mice regarding the degree of HIS re-population.

The second approach is to introduce human genes into NSG/NOG mice or Rag2^−/−^ IL2rγ^null^ mice by transgenesis or knock-in [4], [11], [23]–[29]. However, embryos and embryonic stem (ES) cells derived from NOG/NSG mice are not suitable for transgenesis and knock-in purposes, respectively, due to the lack of genomic information. Currently, the only way to generate transgenic or knock-in NSG/NOG mice is to generate a transgenic or knock-in mouse for each human gene one at a time in a C57BL/6 or 129 background, and then backcross to NSG/NOG mice for at least 10 generations. Because there are so many HLA genes/human cytokine genes to be introduced into NSG/NOG mice, this approach would be extremely laborious, time consuming, and expensive. In this regard, it is noteworthy that human cytokines knock-in Rag2^−/−^ IL2rγ^null^ mice have been generated [23], and this technology solved the backcrossing issue. In the case of human cytokine expression, it is ideal that the level of transgenic expression of the human cytokine genes can be shut off when they are no longer needed, which is difficult to do in a transgenic or knock-in mouse model.

An alternate approach would be to use a viral vector to introduce the human gene of interest. Adeno-associated virus (AAV), a single-stranded virus that belongs to the Parvoviridae family, is characterized by safety, low toxicity, and ability to confer stable expression with very low immunogenicity. Therefore, AAV is considered an interesting candidate vector for gene delivery. AAV infects both dividing and non-dividing cells, and persists in an extra-chromosomal state without integrating into the genome of host cells [30]. In fact, an AAV vector has been used to deliver a gene encoding a monoclonal antibody [31]–[34], and more recently, hepatitis B virus DNA [35]. There are more than 10 serotypes of AAV. In this study, we have chosen AAV serotype 9 (AAV9) because compared to the other serotypes, AAV9 has additional advantages of transducing high degrees of transgenes into the host cells as well as being capable of infecting a wide range of different animal tissues [36].

In the current study, we used a novel AAV9 vector-mediated gene delivery system to introduce human genes encoding HLA-A2 (A2) and selected human cytokines (IL-3, IL-15, and GM-CSF) in an effort to facilitate and improve the repopulation of HIS upon HSCs transplantation. We confirmed that immunizing our HIS mice with adenoviral vaccines expressing antigens of human pathogens successfully induced a high level of antigen-specific A2-restricted human CD8^+^ T-cell responses. Furthermore, A2-restricted CD8^+^ T-cell responses induced in our HIS mice immunized with a human malaria vaccine were shown to exert *in vitro* and *in vivo* cytotoxic activity against malaria antigen-expressing A2-positive hepatocytes.

## Materials and Methods

### Ethics statement

All animal experiments were carried out in strict accordance with the Policy on Humane Care and Use of Laboratory Animals of the United States Public Health Service. The protocol was approved by the Institutional Animal Care and Use Committee (IACUC) at The Rockefeller University (Assurance # A3081-01). CO_2_ was used for euthanasia, and all efforts were made to minimize suffering. Human fetal liver samples were obtained via a non-profit partner (Advanced Bioscience Resources, Alameda, CA) without any information that would identify the subjects from whom they were derived and did not require IRB approval for its use, as previously described [37].

### Mice

NOD.Cg-*Prkdc^scid^ IL2rg^tmWjl^*/Sz (NSG) mice and HLA-A2.1/HHD transgenic NSG (A2-Tg NSG) mice were purchased from The Jackson Laboratories and maintained under specific pathogen-free conditions in the animal facilities at Comparative Bioscience Center of The Rockefeller University.

### Generation of AAV vectors

The HLA-A2.1 (HHD) gene, encoding an interspecies hybrid MHC class I gene [38] (consisting of the alpha1 and alpha2 domains of the HLA-A2.1 gene and alpha3, and the cytoplasmic and transmembrane domains of the murine H-2D^b^ gene), covalently linked to a human β2-microglubulin (hβ2m), was cloned from A2-Tg NSG mice using PCR. HLA-A2.1 is herein designated HLA-A2 or A2 throughout the text. Human GM-CSF, IL-3, IL-4, IL-7, and IL-15 cDNA were purchased from OriGene. Human IL-15 was modified by replacing with the bovine preprolactin signal peptide [39], leading to efficient secretion of human IL-15. Human IL-3, IL-4, IL-7, IL-15, GM-CSF, and HLA-A2 cDNAs were subsequently subcloned into pAAV CMV plasmids (Stratagene La Jolla, CA). For production of AAV9 pseudo-typed vectors, each of the vector genome plasmids was co-transfected with pAAV2/9 (encoding the AAV2 replicase and AAV9 capsid sequence [40]) and the helper plasmid pAdΔF6 (an Ad helper plasmid that provides Ad helper functions of E2, E4, and VA RNA [41]). This resulted in recombinant AAV9 encoding GFP and luciferase (AAV9-GFP-Luc), human GM-CSF (AAV9-GM-CSF), human IL-3 (AAV9-IL-3), human IL-4 (AAV9-IL-4), human IL-7 (AAV9-IL-7), human IL-15 (AAV9-IL-15), and HLA-A2 (AAV9-A2). AAV9 vectors were purified by filtration cascade, followed by iodixanol step gradient centrifugation and titration as described previously [42]. Plasmid pAAV-CMV was used as the standard for quantifying the titer of AAV9 encoding HLA-A2 or AAV9 encoding each of the human cytokines using real-time PCR.

### Evaluation of *in vitro* expression of human cytokines and HLA-A2 mediated by AAV9 vectors

The production of human cytokines, encoded by AAV9 vectors, was determined by infecting a murine macrophage cell line, MC57G (CRL2295TM - American Type Culture Collection, Manassas, VA), with the AAV9 vectors, followed by collecting the culture supernatant and performing ELISAs. Briefly, one day after plating MC57G cells in tissue culture plates, cells were infected with serially diluted AAV9 vectors encoding the respective human genes. Forty-eight hours after infection with AAV9 encoding each of the human cytokines, secretion of GM-CSF, IL-3, IL-4, IL-7, and IL-15 into the culture supernatant was determined using ELISAs [43]. Similarly, 48 hours after infecting MC57G cells with AAV9-A2, the level of A2 expression by the transduced MC57G cells was determined by FACS, using antibodies against HLA-A2 (clone BB7.2 - BioLegend, San Diego, CA) and hβ2m (Clone 2M2 - BioLegend)[35], [44].

### Luciferase expression using noninvasive bioluminescent imaging

NSG mice were injected intrathoracically or i.v. with 1×10^10^ genomic copies (GC) of each of AAV9-GFP-Luc. Two weeks later, luciferase expression in the mice was monitored using Caliper Life LifeSciences IVIS®Lumina/Living Image (Caliper LifeScience, Hopkinton, MA). Briefly, after anesthetizing the mice, 200 µL 15 mg/mL D-luciferin (Gold Biotechnology, St. Louis, MO, USA) was injected i.p., and whole body *in vivo* imaging analyses were performed for 30 sec to 2 min using an *in vivo* imaging system (IVIS®Lumina) as previously described [45].

### Monitoring *in vivo* expression of human cytokines and HLA-A2 mediated by AAV9 vectors

NSG mice were intravenously (i.v.) given 5×10^9^ GC of AAV9-GM-CSF, AAV9-IL-3, AAV9-IL-4, AAV9-IL-7, or AAV9-IL-15. In some experiments, 1×10^9^ GC/mouse of AAV9-GM-CSF was also administered by i.v. Sera were collected after 1, 2, 4, 10, and 16 weeks, and the levels of the respective human cytokines produced in the sera of AAV-transduced NSG mice were determined by using ELISAs (BioLegend). Another groups of NSG mice were administered 5×10^10^ GC by intrathoracically and 5×10^10^ GC by i.v. of AAV9-A2. Four weeks later, the thymus was collected from the injected mice, and co-expression of HLA-A2/hβ2m was determined by FACS and immunohistochemical analyses as described elsewhere in the Materials and Methods section.

### Thymus immunohistochemistry

Immunohistochemical analyses of the thymus collected from AAV9-A2-injected NSG mice were performed as described [46]. Briefly, after collecting thymus from AAV9-A2-injected NSG mice, they were washed and embedded in compound 4583 (Tissue Tek) and frozen in a bath of ethanol and dry ice. Blocks were sliced into 8 to 12 nm thick slices using a microtome (Microm HM500 OM cryostat), and were fixed in 4% formaldehyde solution for 10 min. The slides were blocked for 1 hour with PBS buffer containing a cocktail of 5% normal goat sera and 5% normal mouse sera. After washing three times with PBS, the slides were incubated for overnight at 4°C with the purified anti-mouse CD326 (BioLegend) and anti-HLA-A2.1 antibodies (BioLegend), followed by three washes and incubation for 1 hour at room temperature in the dark with the secondary antibodies Alexa Fluor 555-labeled goat anti-rat IgG (BioLegend) and Alexa Fluor 488-labeled goat anti-mouse IgG (BioLegend), respectively. The slides were washed once and nuclei were counterstained with Hoechst 33342 (Sigma) for 5 min. Finally, the slides were washed three times, mounted with mounting medium (KPL), and examined using an Olympus IX-70 inverted microscope equipped with a camera. Images were acquired at 100× magnification and analyzed using Imaris software version 6.2.

### Determination of AAV9-specific and transgene-specific GC number in selected organs

AAV9-specific and the transgene-specific GC number present in selected organs were determined as previously described [36]. Briefly, various organs, including lung, liver, spleen, kidney and bone marrow, were collected 6 and 20 weeks after NSG mice were given recombinant AAV9 vectors, as described above. Each organ was pretreated with proteinase K prior to being homogenized using a tissue homogenizer. DNA was isolated from the tissue lysate following the DNeasy Blood and Tissue kit protocol (Qiagen, Germantown, MD). After determining total DNA concentration by a spectrophotometry, 100 ng of DNA from each sample was used as a template material for a real-time PCR. The total viral GC number of all the AAV9 vectors present in certain organs was quantified by using SYBR Green PCR Kit (Bio-Rad) with primers specific to the AAV9 vector itself, which were 5′-GGCGGAGTTGTTACGACAT-3′ (forward) and 5′-GGGACTTTCCCTACTTGGCA-3′ (reverse). The GC number of each transgene transduced in certain organs by respective AAV9 vector was quantified with primers specific to the transgenes, i.e. HLA-A2, huGM-CSF, huIL-3 and huIL-15. The primers used for HLA-A2 were 5′-ACATGGAGCTTGTGGAGACC-3′ (forward) and 5′-AGGACACCCAGAACAGCAAC-3′ (reverse). The primers used for huGM-CSF, huIL-3 and huIL-15 were; 5′-CACTGCTGCTGAGATGAATGA-3′ (forward) and 5′-CTGGGTTGCACAGGAAGTTT-3′ (reverse), 5′-TGGGGAAGACCAAGACATTC-3′ (forward) and 5′-CGGAATTCATTCCAGTCACC-3′ (reverse), and 5′-ATGGACAGCAAAGGTTCGTC-3′ (forward) and 5′- ACTTTGCAACTGGGGTGAAC-3′ (reverse), respectively. The standard curve of each AAV9 vector, as GC number control, was generated, using a 7-log spanning serial dilution between 100 pg and 100 fg of DNA in 50 ng/µl salmon sperm DNA. All samples and controls were run in triplicate. From this data set, we obtained the number of viral GC/µg of total genomic DNA for total AAV9 or for each transgene transduced by the recombinant AAV9 vector in selected organs. One picogram of AAV9 DNA is equivalent to 3.6×10^5^ GC.

### Purification of human hematopoietic stem cells (HSCs) and xenogeneic transplantation

Lymphocytes were isolated from fetal liver samples as described elsewhere [6]. CD34^+^ HSCs were isolated from lymphocytes using anti-human CD34^+^ microbeads (Miltenyi Biotec, Germany) according to the manufacturer instructions. HSC purity was evaluated using flow cytometric analyses, and the percentage of CD34^+^ cells was confirmed to be higher than 90%. Young NSG mice (less than 4 weeks old) were transduced with AAV9-A2 and/or AAV9-hucytokines as described above and 2 weeks later, they received 150 cGy total body sub-lethal irradiation for myeloablation. A few hours later, 1×10^5^ human CD34+ cells were transplanted by i.v. infusion to each AAV9-injected, irradiated NSG mouse.

### Phenotypic analyses of human CD45^+^ cells in the blood of AAV9-A2/hucytokines-transduced, HSCs-transplanted NSG mice

The reconstitution status of human CD45^+^ cells in the blood of AAV9-A2/hucytokines-transduced NSG mice was monitored 6, 10, and 14 weeks after HSC transplantation by determining the percentage of human CD45+ cells in the peripheral blood using flow cytometric analyses. Upon lysing red blood cells, PBMCs were purified from peripheral blood collected from the mice. After washing the cells twice, purified PBMCs were blocked for 5 min on ice using normal mouse sera supplemented with anti-CD16/CD32 (clone 93 - BioLegend). Cells were washed once, and stained for 40 min on ice in the dark with the following antibodies: Pacific Blue anti-human CD45 (clone HI30 - BioLegend), PerCP/Cy5.5 anti-mouse CD45 (clone 30-F11 – BioLegend), PE-Cy7 anti-human CD3 (clone UCHT1 - BioLegend), APC-Cy7 anti-human CD4 (clone RPA-T4 - BioLegend), Alexa Fluor 700 anti-human CD8 (clone HIT8a - BioLegend), Alexa Fluor 647 anti-human CD161 (clone HP-3G10 - BioLegend), PE anti-human CD19 (clone HIB19 - BioLegend), and APC anti-human CD3 (clone HIT3a - BioLegend). After staining, cells were washed twice with PBS containing 2% FBS, fixed with 1% paraformaldehyde, and analyzed using a BD LSR II (BD Biosciences).

### Immunization of AAV9-transduced, HSC-transplanted NSG mice with recombinant adenoviral vaccines

Sixteen weeks after HSC engraftment, which coincides with 18 weeks after AAV9 inoculation, AAV9-transduced NSG mice, as well as A2-Tg NSG mice, were immunized with a recombinant adenovirus serotype 5 (Ad5) expressing the circumsporozoite (CS) protein of *Plasmodium falciparum* (AdPfCS)[47] or the p24 antigen of HIV-1 (Adp24)[48]. Briefly, the mice were immunized intramuscularly (i.m.) with 5×10^10^ viral particle units (vp) of each AdPfCS and Adp24. Two weeks later, the level of the A2-restricted CD8+ T-cell response specific for PfCS and HIV-p24 antigens was determined using an IFN-γ ELISpot assay and ICCS assay as described below.

### Vaccine-induced A2-restricted human CD8^+^ T-cell response in AAV9-transduced, HSC-transplanted NSG mice

Two weeks after vaccination with AdPfCS and Ad-p24, spleens and liver were harvested from corresponding immunized AAV9-transduced, HSC-transplanted NSG mice. Lymphocytes were also processed from respective organs. After isolation of lymphocytes, the cells were counted and used for IFN-γ ELISpot and ICCS assays upon stimulation with synthetic peptides corresponding to the A2-restricted CD8^+^ T-cell epitopes of the PfCS protein (1-YLNKIQNSL; 2- KLRKPKHKKL; 3- SLKKNSRSL)[49] and HIV-p24 antigen (TLNAWVKVV)[50]. For ELISpot assays, the relative numbers of PfCS antigen-specific and HIV-p24 antigen-specific IFN-γ-secreting human CD8^+^ T cells among lymphocytes obtained from spleen and liver of Ad-immunized HIS mice were determined using ELISpot assays as previously described [47]–[49]. For ICCS assays, lymphocytes were stimulated for 4–6 hours using a pool of the synthetic peptides listed above or PMA-ionomycin (as a positive control) in the presence of brefeldin at 37°C with 5% CO_2_. ICCS assays were performed as previously described [50]. Briefly, after blocking with the anti-mouse CD16/CD32 antibody, lymphocytes were stained for surface markers with antibodies against CD45, CD3, and CD8. Next, lymphocytes were permeabilized with perm/wash solution (BD Biosciences), stained with the FITC-labeled anti-human IFN-γ antibody, fixed with 1% paraformaldehyde, and analyzed using a BD LSR II (BD Biosciences).

### *In vivo* and *in vitro* cytotoxic activity of vaccine-induced A2-restricted human CD8^+^ T cells

For *in vitro* cytotoxic assays, livers from naïve HIS mice (NSG mice transduced with AAV9-A2/hucytokines followed by HSC transplantation) were perfused and hepatocytes were collected, as described [51]. After labeling hepatocytes with 6 µM carboxyfluorescein diacetate succinimidyl ester (CFSE; Molecular Probes, Eugene, OR) for 15 min at 37°C [52], they were pulsed with peptides corresponding to the A2-restricted CD8^+^ T cell epitopes listed above at 2 µg/ml for 1 hour. CFSE-labeled naïve hepatocytes pulsed with peptide were then mixed with the same volume (100 µl) of various numbers of CD8^+^ enriched T cells from AdPfCS-vaccinated HIS mice (as effector cells) in conical polypropylene Costar cluster tubes (Costar, Corning, NY). The cell mixture was centrifuged at low speed (200 rpm) for 1 min and incubated at 37°C in 5% CO_2_ incubator for the indicated times (0.5 to 5 hours). The cells were washed with culture media containing 1% BSA at room temperature, and either fixed with 1% PFA or permeabilized with Cytofix/Cytoperm solution (BD Biosciences). Next, the cells were washed twice with staining buffer (D-PBS, 1% BSA, 0.1% saponin) and resuspended in staining buffer. After incubation for 1 hour on ice with the biotinylated anti-cleaved caspase 3 monoclonal antibody (BD Biosciences, Mississauga, ON) [53], [54], cells were incubated for 30 min on ice with strepavidin-APC (Sigma, St. Louis, MO) and analyzed using a BD LSR II (BD Bioscience).

For *in vivo* cytotoxic assays, a group of 8 HIS mice were immunized i.m. with 5×10^10^ vp of AdPfCS. Two weeks later, four AdPfCS-immunized, as well as four naïve HIS mice, received 50 µg of a plasmid DNA encoding the PyCS or PfCS gene [45] via hydrodynamic tail vein (HTV) injection less than 5 seconds after the plasmid DNA was diluted in PBS in a total volume of 2 ml [45], [55]. Three days after the HTV injection, total RNA was extracted from liver samples. Purified RNA was transcribed to cDNA using MultiScribe Reverse Transcriptase (Applied Biosystems, Foster City, CA) following the manufacturer's instructions. To perform real-time PCR to evaluate expression of PfCS protein-specific mRNA, we designed a specific set of primers and a Taqman probe: ACC CTA ATG CCA ATC CCA AC (F), CCC GTT GCC TTG ATT GTT C (R), and 56-FAM/ACC CTA ATG/ZEN/CTA ACC CGA ACG CC/3IABkFQ (P). We also designed a set of primers and a Taqman probe for PyCS protein-specific mRNA: TGA GGG TGA GGA AGA GGA AG (F), CTG TTG CTC ACG ATG TTG AAG (R), and 56-FAM/TGA ACA AGC/ZEN/AGC CCG AGA ACC T/3IABkFQ (P). Finally, mouse GADPH Endogenous Control (VIC®/MGB Probe, Primer Limited) was purchased from Applied Biosystems. Real-time PCR was performed using an ABI Prism 7500 Fast Sequence Detector (Applied Biosystems) as previously described [47], [48]. The copy number for the respective mRNAs in each sample was normalized to the GAPDH copy number. Each value was expressed as the mean of the ratio of PfCS protein or PyCS protein mRNA relative to GAPDH mRNA from duplicate samples of two independent reactions.

## Results

### Construction of AAV9 vectors encoding human cytokines and HLA-A2, and evaluation of transgene production and expression *in vitro*

**Figure 1:**
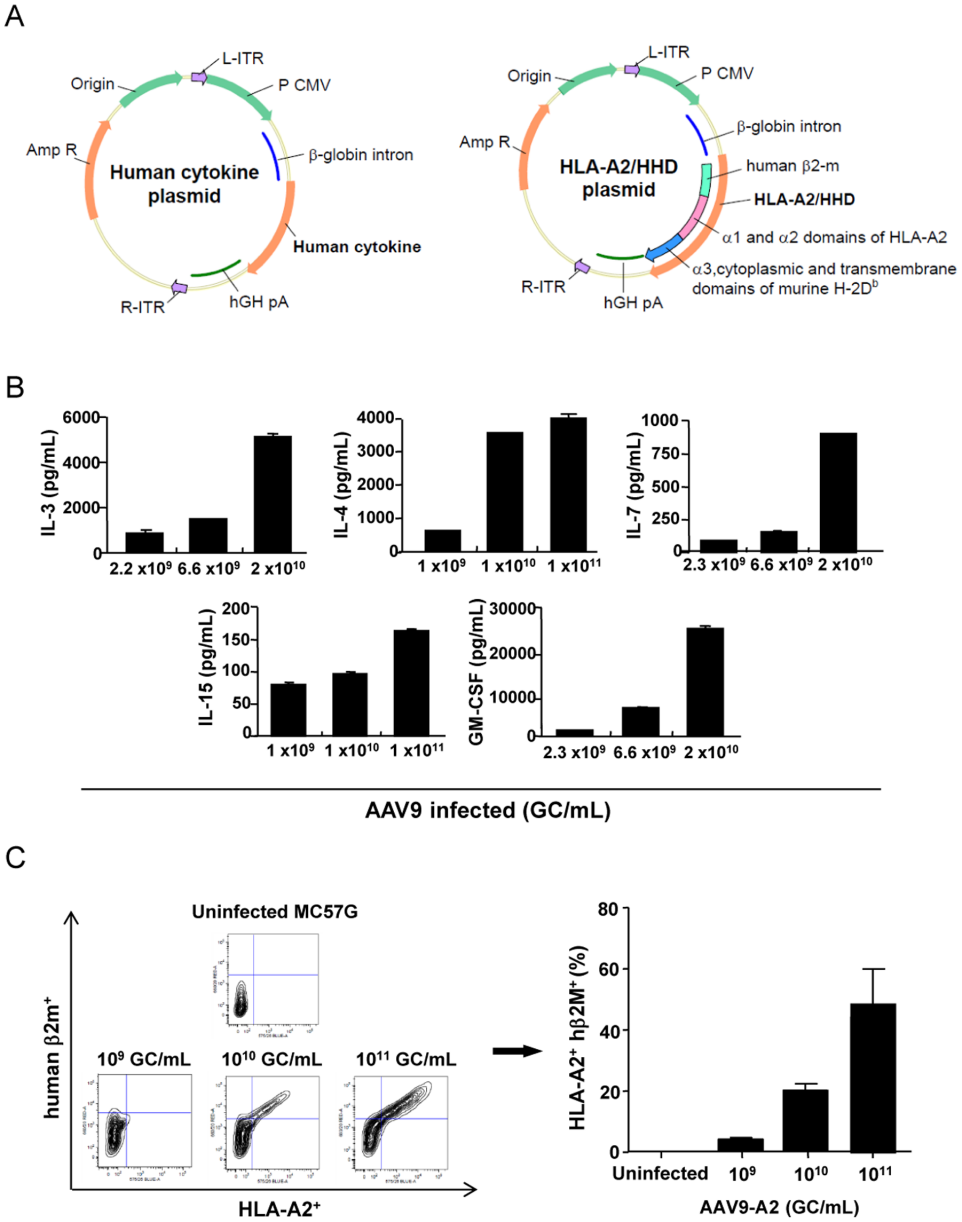
*In vitro* expression of human cytokines and HLA-A2.1 in MC57G cells. (A) Maps of Zac2.1 plasmids modified to encode human cytokines and HLA-A2.1 (HHD) containing α1 and α2 domains of HLA-A2.1, α3, cytoplasmic and transmembrane domains of murine H-2D^b^, and hβ2m, are shown. These plasmids were used to construct AAV9 viral particles. (B) MC57G cells were infected *in vitro* with AAV9 encoding the respective cytokine, and the production of each cytokine was determined using ELISA. (C) MC57G cells were infected *in vitro* with different doses (1×10^9^, 1×10^10^, or 1×10^11^ GC/mL) of AAV9-encoding HLA-A2.1/hβ2m (AAV9-A2). Expression of HLA-A2.1 and hβ2m was evaluated using flow cytometric analyses.

We constructed recombinant AAV9 vectors encoding human cytokine genes including IL-3, IL-4, IL-7, IL-15, and GM-CSF, as well as those encoding HLA-A2 (Figure 1A). All these human cytokines are shown to be necessary for a proper human immune system development and function in NSG/NOG mice [10], [11]. To confirm the production and expression of human cytokines and A2, respectively, encoded by AAV9, we infected a murine macrophage cell line, MC57G, with various concentrations of each AAV9 *in vitro*. We found that MC57G cell lines infected with the corresponding AAV9 produced significant amounts of human IL-3, IL-4, IL-7, IL-15, and GM-CSF in culture in a dose-dependent manner (Figure 1B). HLA-A2 and hβ2m co-expression was also detected upon infection of MC57G cells with AAV9-A2, which encodes chimeric A2/hβ2m, in a dose-dependent fashion (Figure 1C).

### *In vivo* production of human cytokines in NSG mice upon transduction with AAV9 vectors

**Figure 2:**
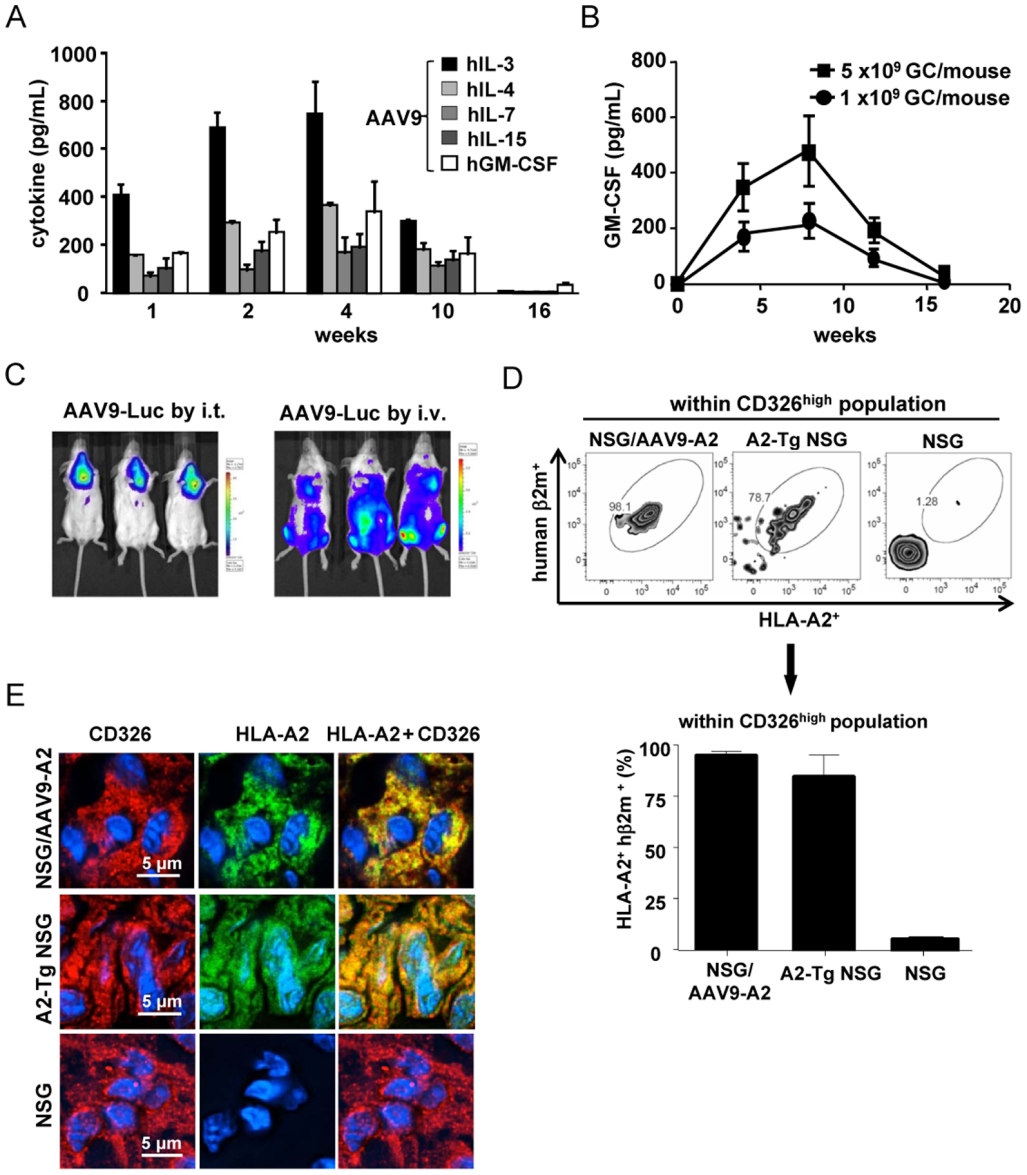
*In vivo* expression of human cytokines and HLA-A2/hβ2-m in NSG mice upon AAV9-mediated gene delivery. (A) NSG mice were inoculated with 5×10^9^ GC/mouse of AAV9 encoding each cytokine, and 1, 2, 4, 8, 10, or 16 weeks later, sera were collected from the mice and cytokine production was determined using ELISA. (B) The level of human GM-CSF produced in the sera was determined after inoculation of NSG mice with a high (5×10^9^ GC/mouse) or a low (1×10^9^ GC/mouse) dose of AAV9-GM-CSF. (C) Luciferase expression 2 weeks after inoculation of NSG mice with 1×10^10^ GC of AAV9-GFP-Luc via intrathoracic or i.v. route is shown by injecting D-luciferin intraperitoneally, followed by whole body *in vivo* imaging analyses. (D) NSG mice were administered intrathoracically with 5×10^10^ GC of AAV9-A2 and 4 weeks later, the expression of HLA-A2 and hβ2m by CD326^HIGH^ cells within the thymus of AAV9-A2-infected NSG mice, A2-Tg NSG mice, and naïve NSG mice was determined using flow cytometric analyses. (E) Immunohistochemical analyses show HLA-A2 (green) and CD326 (red) staining of thymic tissue from AAV9-A2-transduced NSG mice, A2-Tg NSG mice, and naïve NSG mice. Hoechst 33342 (blue) was used to counterstain nuclei.

To determine the level of *in vivo* production of human cytokines in NSG mice transduced by AAV9 vectors encoding the human cytokines, each young (3- to 4-week-old) NSG mouse was intravenously administered 5×10^9^ GC of AAV9 encoding each human cytokine. After 1, 2, 4, 10, and 16 weeks, sera were collected from the mice and the level of each human cytokine produced was measured using ELISA. As shown in Figure 2A, the level of all human cytokines was detectable up to 10 weeks after inoculation with AAV9 vectors. The production levels of all cytokines tested formed a bell-shaped curve with peaks at 4 weeks, and the cytokine levels became almost undetectable at week 16, except for human GM-CSF. When NSG mice were inoculated with two different doses of AAV9-GM-CSF, high (5×10^9^ GC/mouse) and low (1×10^9^ GC/mouse), and the level of human GM-CSF secreted in the sera was monitored, we observed peak secretion 8 weeks after NSG mice were inoculated with either dose of AAV9-GM-CSF (Figure 2B). More importantly, upon injection with 1×10^9^ GC of AAV9-GM-CSF, the level of human GM-CSF became undetectable 16 weeks post AAV9-GM-CSF inoculation (Figure 2B).

### *In* vivo expression of A2/hβ2m in NSG mice upon transduction with the AAV9 vector

To determine the tissue tropism of AAV9 vectors, NSG mice were first intrathoracically or i.v. inoculated with 1×10^10^ GC/mouse of AAV9 encoding GFP-luciferase. Two weeks later, non-invasive whole-body bioluminescent imaging analyses were performed. As shown in Figure 2C, intrathoracic administration of the AAV9 vector resulted in a localized expression of the transgene in the thoracic region, whereas i.v. injection led to a systemic expression of the transgene. Next, we aimed to examine the *in vivo* expression of both HLA-A2 and hβ2m molecules in NSG mice inoculated with an AAV9-A2 vector that encodes both molecules compared with A2-Tg NSG mice and naïve NSG mice. For this purpose, we inoculated each NSG mouse intrathoracically with 5×10^10^ GC and i.v. with 5×10^10^ GC of AAV9-A2 vector. Four weeks later, thymus was removed from the AAV9-A2-inoculated NSG mice, A2-Tg NSG mice, and naïve NSG mice. After obtaining a single suspension, A2/hβ2m expression was evaluated. Flow cytometric analyses of thymus-derived lymphocytes showed a high level of expression of both A2 and hβ2m molecules within the CD326^high^ epithelial cell population, and the level was similar or higher than that observed in HLA-A2 transgenic NSG mice (Figure 2D). A2/hβ2m expression was confirmed by immunohistochemistry (IHC), in which the level of expression of HLA-A2 in the thymus of AAV9-A2-inoculated NSG mice appeared comparable to A2-Tg NSG mice (Figure 2E). The non-leukocyte (CD45^−^) fraction in other tissues, such as liver, spleen, and bone marrow, also showed high expression of A2/hβ2m after AAV9-A2 transduction at 20 weeks after AAV9-A2 inoculation (Figure S1). This is particularly important considering that hepatocytes derived from AAV9-A2-inoculated NSG mice can present A2-restricted peptides to PfCS-specific A2-restricted human CD8^+^ T cells, which then should be able to recognize and lyse the hepatocytes.

### *In vivo* persistence of AAV9 and transgene GC in NSG mice upon AAV9 injection

**Figure 3:**
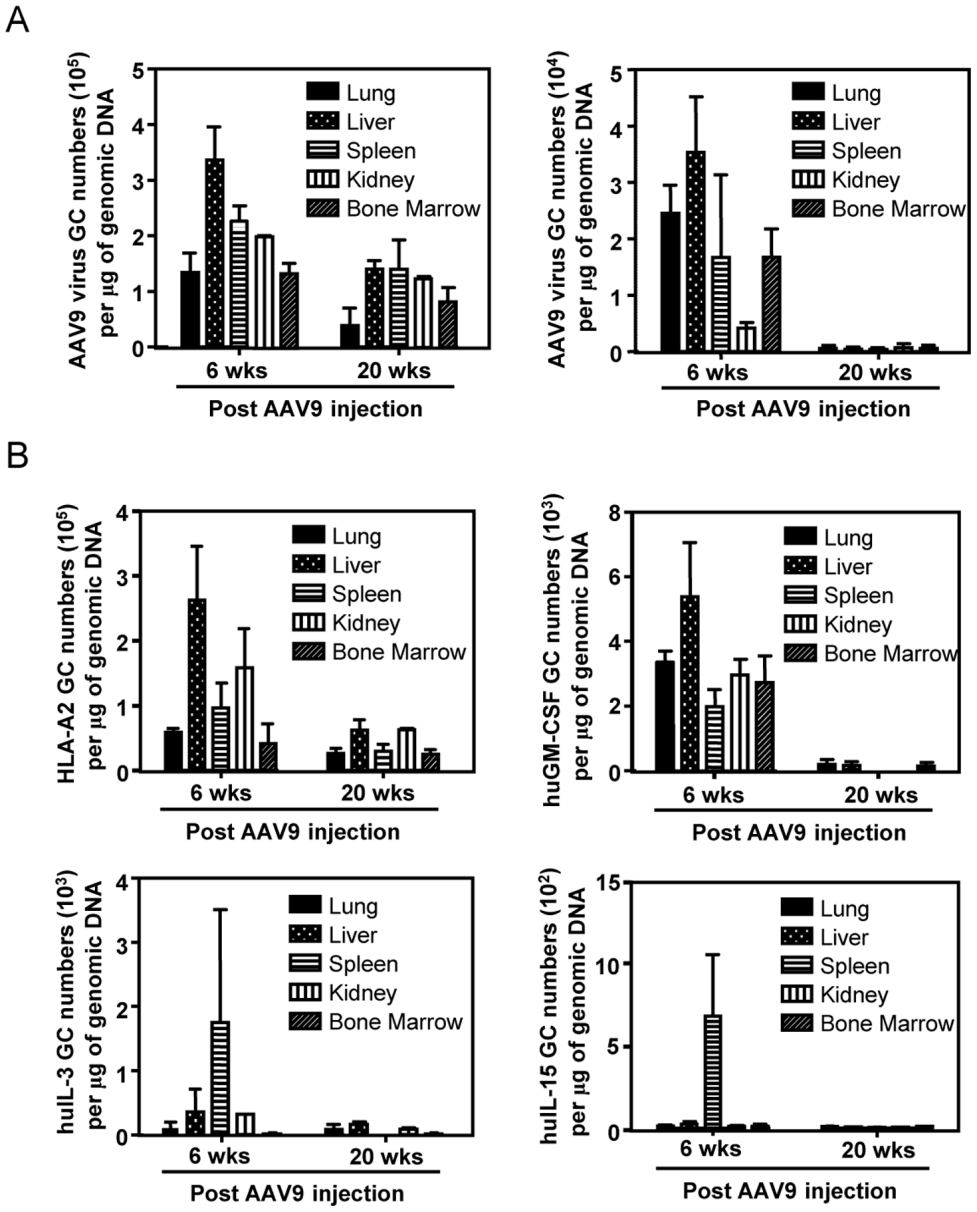
GC number specific for AAV9 and the transgenes present in selected organs of NSG mice at 6 weeks and 20 weeks after AAV9 injection. Lung, liver, spleen, kidney and bone marrow, were collected 6 and 20 weeks after NSG mice were given recombinant AAV9 vectors (5×10^9^ GC of AAV9-GM-CSF, AAV9-IL-3, and AAV9-IL-15 by i.v., or 1×10^11^ GC of AAV9-A2 by i.v. and intrathoracically), and DNA was isolated from the respective organs. (A) The number of AAV9 vector GC was determined by using a set of primers specific to AAV9 vector. (B) The number of GC specific for the transgenes - HLA-A2, hGM-CSF, hIL-3 and hIL-15 - was determined by using a set of primers to the respective transgene. There was a low number (10–10^3^ GC; depending on the transgene) of GC detected in naïve NSG mice, as a non-specific background, and, therefore, this background GC number was subtracted from the GC number in experimental groups.

To determine the AAV9 vector and respective transgene GC numbers, lungs, liver, kidney, spleen and bone marrow were isolated from NSG mice 6 and 20 weeks after recombinant AAV9 vector administration. AAV9- and transgene-specific GC numbers were determined from DNA isolated from the organs by qPCR using AAV9- and transgene-specific primers, respectively. As shown in Figure 3A, the mice that received a high dose (1×10^11^ GC) of AAV9-A2 maintained relatively high AAV9 vector GC numbers at 20 weeks post injection, whereas, the GC number in mice that received a low dose (1.5×10^10^ GC) of AAV9-hucytokines cocktail (AAV9-GM-CSF, AAV9-IL-3 and AAV9-IL-15) rapidly decreased and was almost undetectable at 20 weeks post injection. When the transgene-specific GC numbers were determined, we observed a similar trend, i.e. only the HLA-A2 GC number remained high (>10^4^ GC) upon a high dose of AAV-A2 injection, while all other cytokine-specific GC numbers became low (<10^3^ GC) in all the organs tested 20 weeks after injection with a low dose of a cocktail of recombinant AAV9 vectors encoding hucytokines (Figure 3B).

### Reconstitution of human leukocytes from HSCs transplanted in NSG mice facilitated by AAV9-hucytokines or AAV9-A2 transduction

**Figure 4:**
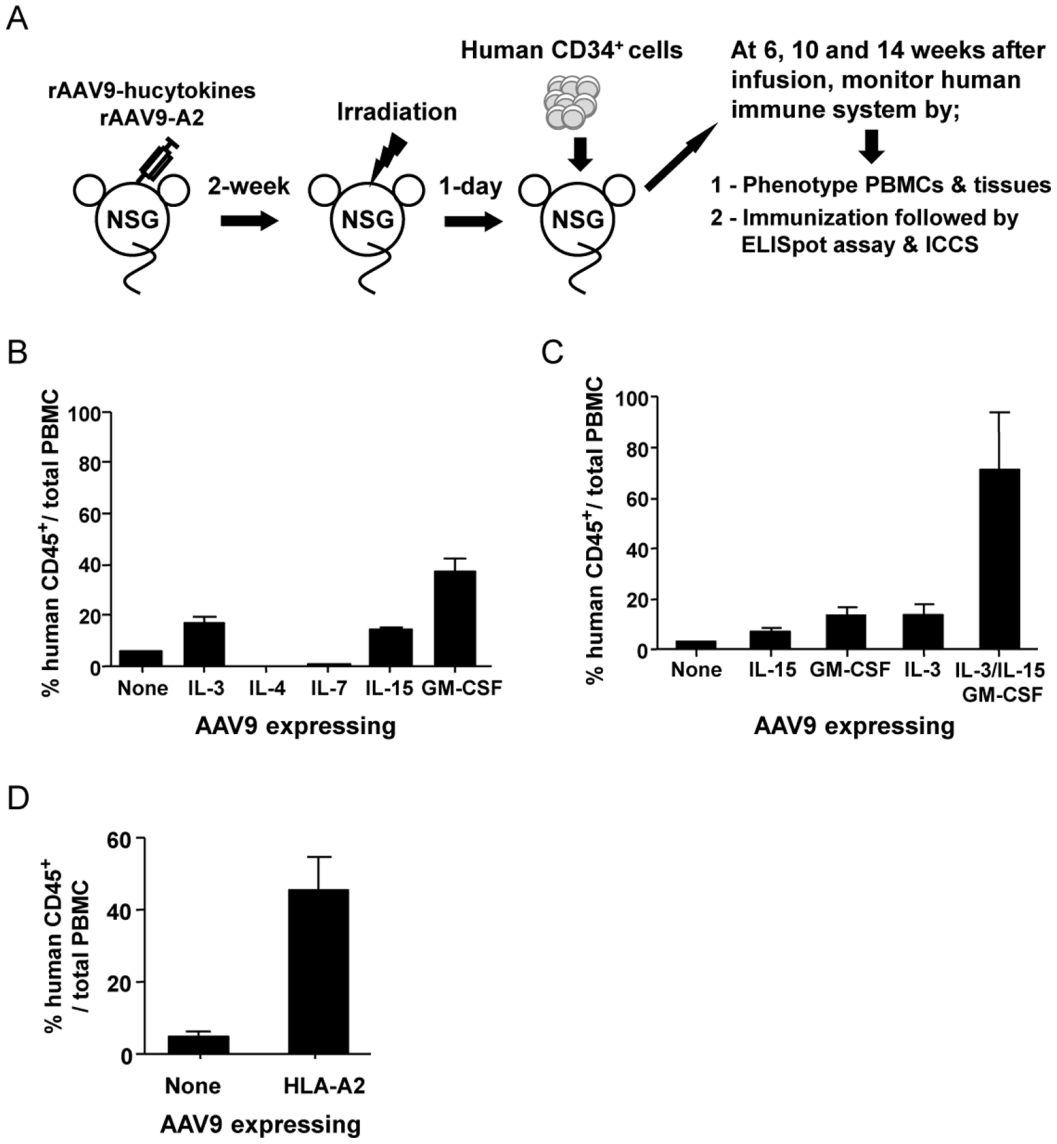
Human leukocyte reconstitution in the peripheral blood of NSG mice transduced with AAV9-hucytokines or AAV9-A2. (A) Schematic representation of the strategic methodology for engrafting human CD34^+^ cells in AAV9-transduced NSG mice. NSG mice were inoculated with AAV9-hucytokines or AAV9-A2 and 2 weeks later, mice were irradiated to myeloablate mouse immune cells. The next day, mice were transplanted i.v. with 1×10^5^ human CD34^+^ cells previously isolated from human fetal liver. (B) The level of human CD45^+^ cell reconstitution in the blood was determined using flow cytometric analyses 10 weeks after engrafting human CD34^+^ cells into NSG mice transduced with AAV9 encoding human IL-3, IL-4, IL-7, IL-15, or GM-CSF. (C) The level of human CD45^+^ cell reconstitution in the blood was determined using flow cytometric analyses 10 weeks after engrafting human CD34^+^ cells into NSG mice transduced with individual or combination of AAV9 encoding selected human cytokines, IL-3 (5×10^9^ GC), IL-15 (5×10^9^ GC), and GM-CSF (1×10^9^ GC). (D) The level of human CD45^+^ cell reconstitution in the blood was determined using flow cytometric analyses 10 weeks after engrafting human CD34^+^ cells into NSG mice transduced with AAV9-A2.

There are several human cytokines that facilitate the development of the human hematopoietic cell lineage, including human IL-3, IL-4, IL-7, IL-15, and GM-CSF. Therefore, to determine the contribution of each human cytokine on the repopulation of human CD45^+^ cells in NSG mice, NSG mice were first transduced with AAV9 encoding the respective human cytokine separately, followed by transduction with a cocktail of these AAV9 vectors. One to two weeks after transduction, NSG mice were sub-lethally irradiated to myeloablate the remaining murine immune cells, followed by an i.v. infusion of 1×10^5^ HSCs, as identified by human CD34^+^ cells (Figure 4A). Thereafter, the percentages of human CD45^+^ cell repopulation in the peripheral blood of AAV9-transduced NSG mice were monitored up to 14 weeks after HSC transplantation.

Among the human cytokines tested, the highest percentage of human CD45^+^ cell reconstitution was observed in NSG mice injected with AAV9 expressing human GM-CSF (40%), IL-3 (20%), and IL-15 (18%)(Figure 4B). The AAV9 encoding these three human cytokines were thus combined and injected into NSG mice at a fixed viral dose (1**×**10^9^ GC/mouse for AAV9 encoding GM-CSF and 5**×**10^9^ GC/mouse for AAV9 encoding IL-3 and IL-15). We observed an additive/synergistic effect of human GM-CSF, IL-3, and IL-15 on the engraftment of human CD45^+^ cells in NSG mice when these cytokines were combined, resulting in approximately 70% of human CD45^+^ cells 14 weeks after the human CD34^+^ cell infusion (Figure 4C). Delivery of the A2/hβ2m genes via intrathoracic and i.v. inoculation of AAV9 vectors to NSG mice also greatly improved the reconstitution of human CD45^+^ cells in peripheral blood mononuclear cells (PBMCs) 14 weeks after the human CD34^+^ cell engraftment (Figure 4D).

### Improved human leukocyte repopulation from HSCs transplanted in NSG mice transduced with AAV9-A2/hucytokines

**Figure 5:**
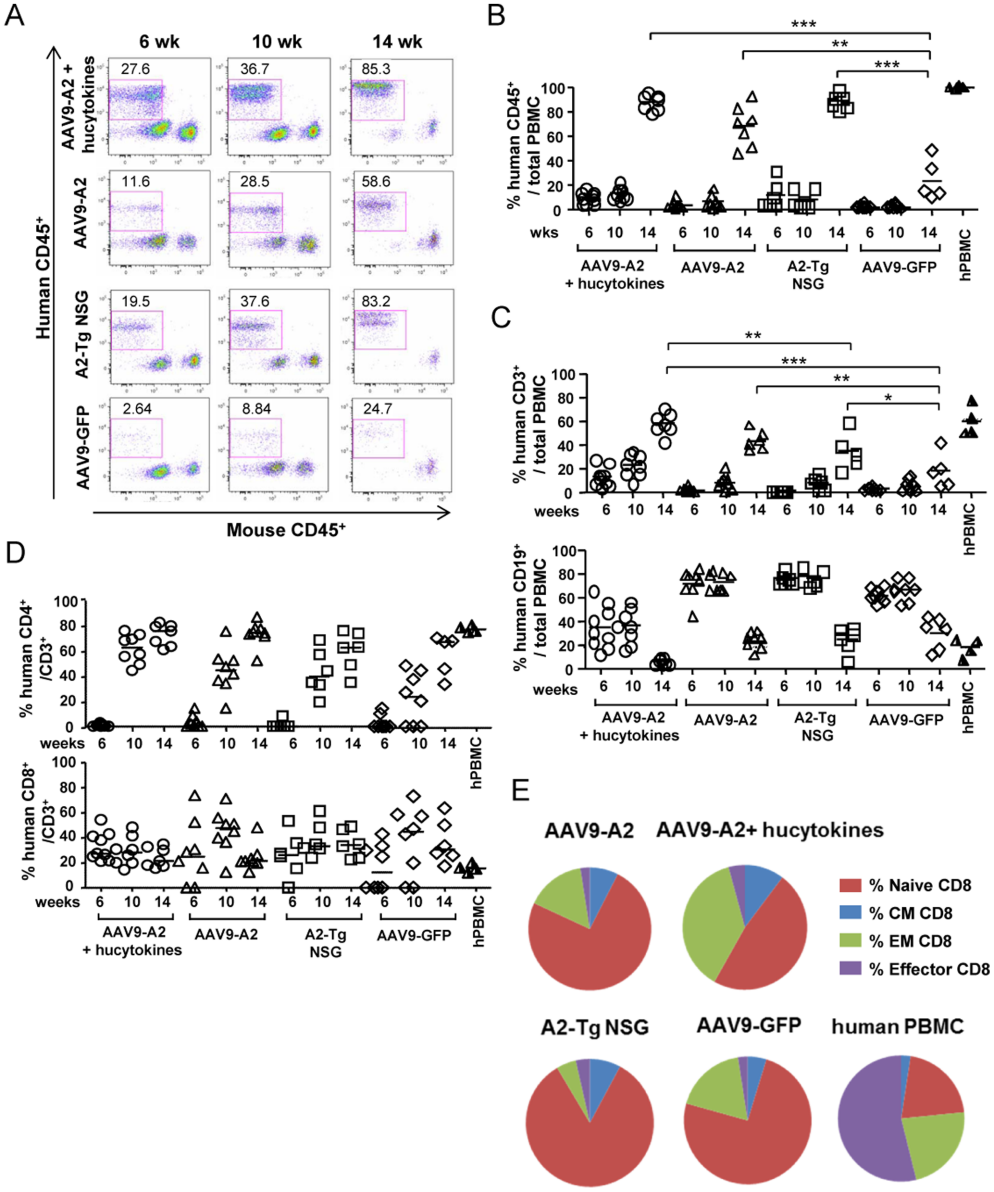
Reconstitution of a human immune system in NSG mice transduced with AAV9-A2/hucytokines. (A) Flow cytometric analyses were performed to determine the level of human CD45^+^ cell reconstitution in the blood of various groups of mice: AAV9-A2/hucytokines-transduced NSG mice, AAV9-A2-transduced NSG mice, A2-Tg NSG mice, or AAV9-GFP-transduced NSG mice 6, 10, or 14 weeks after engraftment of human CD34^+^ cells. (B) The level of human CD45^+^ cell reconstitution in the blood of various groups of NSG mice was determined using flow cytometric analyses 6, 10, or 14 weeks after engrafting human CD34^+^ cells. (C) Percentages of human CD3^+^ T cells and CD19^+^ B cells within the human CD45^+^ cells in the blood of various groups of HIS mice were determined 6, 10, and 14 weeks after HSC engraftment. (D) Percentages of human CD8^+^ and CD4^+^ T cells within the human CD3^+^ T cells in the blood of various groups of HIS mice were determined 6, 10, and 14 weeks after HSC engraftment. (E) The pie charts show the percentage of various human lymphocyte subsets, including naïve, central memory (CM), effector memory (EM) and effector CD8+ T cells, within the human CD45^+^ cells in the blood of various groups of HIS mice determined 14 weeks after HSC engraftment, compared to those in PBMCs of a healthy human subject. * indicates p values <0.05, ** indicates p values <0.01, and *** indicates p values <0.001.

To evaluate whether transduction of HLA-A2 with or without co-transduction of the combined human cytokines could further facilitate reconstitution of human CD45^+^ cells, NSG mice were inoculated with 1×10^11^ GC of AAV9-A2 (5×10^10^ GC each by intrathoracically and i.v.), together with AAV9-hucytokines (5×10^9^ GC of AAV9-IL-3 and AAV9-IL-15, and 1×10^9^ GC of AAV9-GM-CSF by i.v.), or AAV9-A2 alone. A2-Tg NSG mice and NSG mice inoculated with 2×10^10^ GC/mouse of AAV9 encoding GFP-Luc (mock treatment) were also evaluated for the percentage of human CD45^+^ cells in their PBMCs 6, 10, and 14 weeks after HSC transplantation (Figure 5A). Although the human CD45^+^ cell percentage (Figure 5A and B) and absolute numbers (data not shown) in the blood increased over time in all groups, the highest percentage of human CD45^+^ repopulation was found in NSG mice who received both AAV9-A2 and AAV9-hucytokines, as well as in A2-Tg NSG mice. The percentage of human leukocyte population could exceed 75% among total leukocytes in the blood of all mice (Figure 5B).

We next determined the percentages of various subpopulations, including CD19^+^ B cells, CD3^+^ T cells, CD4^+^, and CD8^+^ T cells, among the human CD45^+^ cells using flow cytometric analyses (Figure S2) in the blood (Figure 5C and D) and in the spleen and bone marrow (Figure S3). In the blood, we found that the percentage of CD3^+^ T cells among human CD45^+^ cells was highest in NSG mice inoculated with AAV9-A2 and AAV9-hucytokines (AAV9-A2/hucytokines), which was similar to that observed in human PBMCs (Figure 5C). In contrast, the percentage of CD19^+^ B cells was lowest in this group (Figure 5C). The percentages of both CD4^+^ and CD8^+^ T cells in NSG mice inoculated with AAV9-A2/hucytokines were almost superimposable to those in human PBMCs (Figure 5D). In the spleen and bone marrow, the same trend was observed for CD19^+^ B and CD3^+^ T cells (Figure S3B and C) as in blood. It is noteworthy that the percentage of CD34^+^HLA-A2^+^ cells engrafted in the bone marrow of AAV9-A2/hucytokines-transduced NSG mice was much higher than that in other groups (Figure S4), confirming the superiority of the combination of cytokines for stem cell engraftment compared to other groups having no cytokines. When the absolute numbers of CD4^+^ and CD8^+^ T cells in the blood were determined, the absolute number of both CD4^+^ and CD8^+^ T cells in the blood of NSG mice who received AAV9-A2/hucytokines was much higher than NSG mice that received AAV9-A2 alone or A2-Tg NSG mice (Figure S3D and E). We have also compared the reconstitution status of other immune cell types, including DCs macrophages, and NK cells in different HIS mouse groups, as shown in Table 1. In general, there is no significant difference with regards to the percentage of DCs, macrophages and monocytes, whereas the percentage of NK cells appears to be higher in AAV9-A2/hucytokines-transduced NSG mice compared to other groups of NSG mice. The quality and the function of these cell subsets remain to be investigated. Lastly, when we determined the status of CD8^+^ T cell memory/activation, we found that the percentage of effector memory CD8^+^ T cell subset significantly increased among total CD8^+^ T cells in NSG mice injected with AAV9-A2/hucytokines, compared to those injected with AAV9-A2 alone or A2-Tg NSG mice (Figure 5E). A majority of CD8^+^ T cells in PBMCs from a healthy human subject was found to be effector CD8^+^ T cells (Figure 5E).

**Table 1:** 

Treatment	Mice ID#	Human CD45%/Total PBMCs	huCD3 T%	huCD19 B%	huCD8 T%	huCD4 T%	huNK %	huCD11c DC%	huCD14/CD11b MAC%	huCD14 Mono%
			/Total human CD45+ cells		/T cells		/Total human CD45+ cells			
AAV9-A2	582	68.8	38.2	53.7	30.3	63	3.1	0.20	1.23	0.20
	342	66.4	50.1	40.6	54.9	41.9	2.5	0.54	0.71	0.35
	345	66.8	43.8	48.1	17.3	79.9	4.5	0.41	1.45	0.27
	591	70.3	53.5	36.4	36.7	58.8	3.9	0.48	0.62	0.48
AAV9-A2 + AAV-hucytokines	334	92.7	66.7	16	31.5	63.7	10.1	0.25	0.16	0.27
	350	88.9	58.3	18.7	20.7	74	10.9	0.34	0.36	0.29
	361	88.4	61.3	30.6	29.4	65.6	7.1	0.39	0.44	0.42
	599	82.4	45.5	31.9	35.3	59.7	8.7	0.21	1.09	0.63
A2-Tg NSG	698	68	33.6	59.4	23.2	74	1.8	0.70	2.26	0.43
	368	81.6	26.7	67.4	36.9	59.7	1.3	0.35	1.56	0.20
	369	39.2	29.8	63.3	33	62.5	0.9	0.68	1.52	0.30
	696	78.4	36.9	57.6	45.2	49.7	1.2	0.64	1.65	0.50
	697	81.3	39.8	53.1	38.4	58.4	1.7	0.58	1.46	0.35
AAV9-GFP	330	49.7	28.3	68.4	32.1	62.3	4	0.40	1.15	0.22
	700	58.6	31.4	53.4	32.5	59.2	3.5	0.41	1.75	0.23

### Induction of vaccine-specific A2-restricted human CD8^+^ T-cell response in AAV9-A2/hucytokines-transduced, HSC-transplanted NSG mice

**Figure 6:**
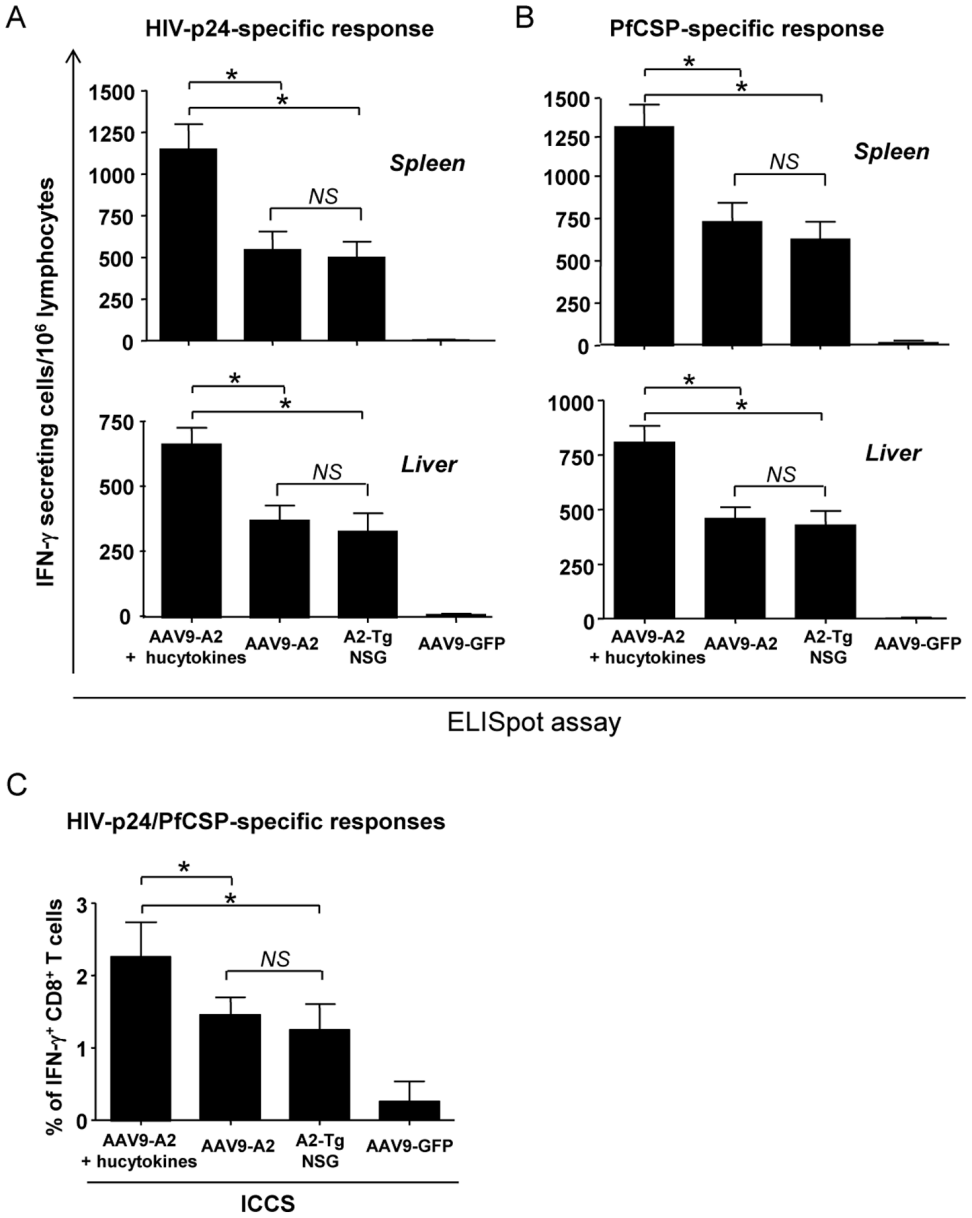
Magnitude of CD8^+^ T cell response induced in AAV9-A2/hucytokines transduced HIS mice immunized with Ad-p24 and AdPfCS. Two weeks after NSG mice were transduced with either AAV9-A2/hucytokines, AAV9-A2, or AAV9-GFP (mock control), these NSG mice, as well as age-matched A2-Tg NSG mice, were engrafted with HSCs. Sixteen weeks after HSCs engraftment, all groups of NSG mice were immunized with Ad-p24 and AdPfCS, and 2 weeks later, splenocytes and liver mononuclear cells were collected. In (A) and (B), ELISpot assays were performed using splenocytes and liver mononuclear cells derived from respective mice in the presence or absence of peptides corresponding to A2-restricted CD8^+^ T-cell epitopes of HIV-p24 (A) and PfCS (B). (C) Intracellular cytokine staining (ICCS) was performed on splenocytes using a pool the peptides corresponding to A2-restricted CD8^+^ T-cell epitopes of HIV-p24 and PfCS. IFN-γ expression was measured in both assays. The results are expressed as IFN-γ-secreting cells/million lymphocytes for ELISpot and as the percentage of IFN-γ^+^ CD8^+^ T cells for ICCS. * indicates p values <0.05. NS, not significant.

In view of the best reconstitution status of various human lymphocytes found in AAV9-A2/hucytokines-transduced NSG mice upon HSC engraftment, we sought to determine the human CD8^+^ T-cell response in these HIS mice compared to AAV9-A2-transduced NSG mice or A2-Tg mice engrafted with HSCs. Mice were immunized with a recombinant adenovirus serotype 5 expressing PfCSP (AdPfCS) or p24 of HIV-1 (Ad-p24). Two weeks later, lymphocytes were isolated from spleen and liver of vaccinated HIS mice, and the relative number and percentage of human T cells secreting IFN-γ in response to synthetic peptides corresponding to the A2-restricted CD8^+^ T cell epitopes of the respective antigen, was determined using ELISpot and intracellular cytokine staining (ICCS) assays. We observed a significantly higher level of A2-restricted human CD8^+^ T-cell responses against the HIV antigen (Figure 6A) as well as the human malaria antigen (Figure 6B) in HSC transplanted NSG mice transduced with both AAV9-A2 and AAV9-hucytokines than in NSG mice receiving AAV9-A2 or in A2-Tg NSG mice, based on ELISpot assays. There was no difference between the latter two groups (p>0.05). A similar trend was observed when we performed ICCS assays (Figure 6C). As expected, we did not observe a significant level of antigen-specific, A2-restricted human CD8^+^ T cell response in NSG mice received AAV9-hycytokines alone, followed by HSC engraftment (data not shown).

### *In vivo* and *in vitro* cytotoxic activity of vaccine-induced A2-restricted human CD8^+^ T cells in AAV9-A2/hucytokines-transduced, HSC-transplanted NSG mice

**Figure 7:**
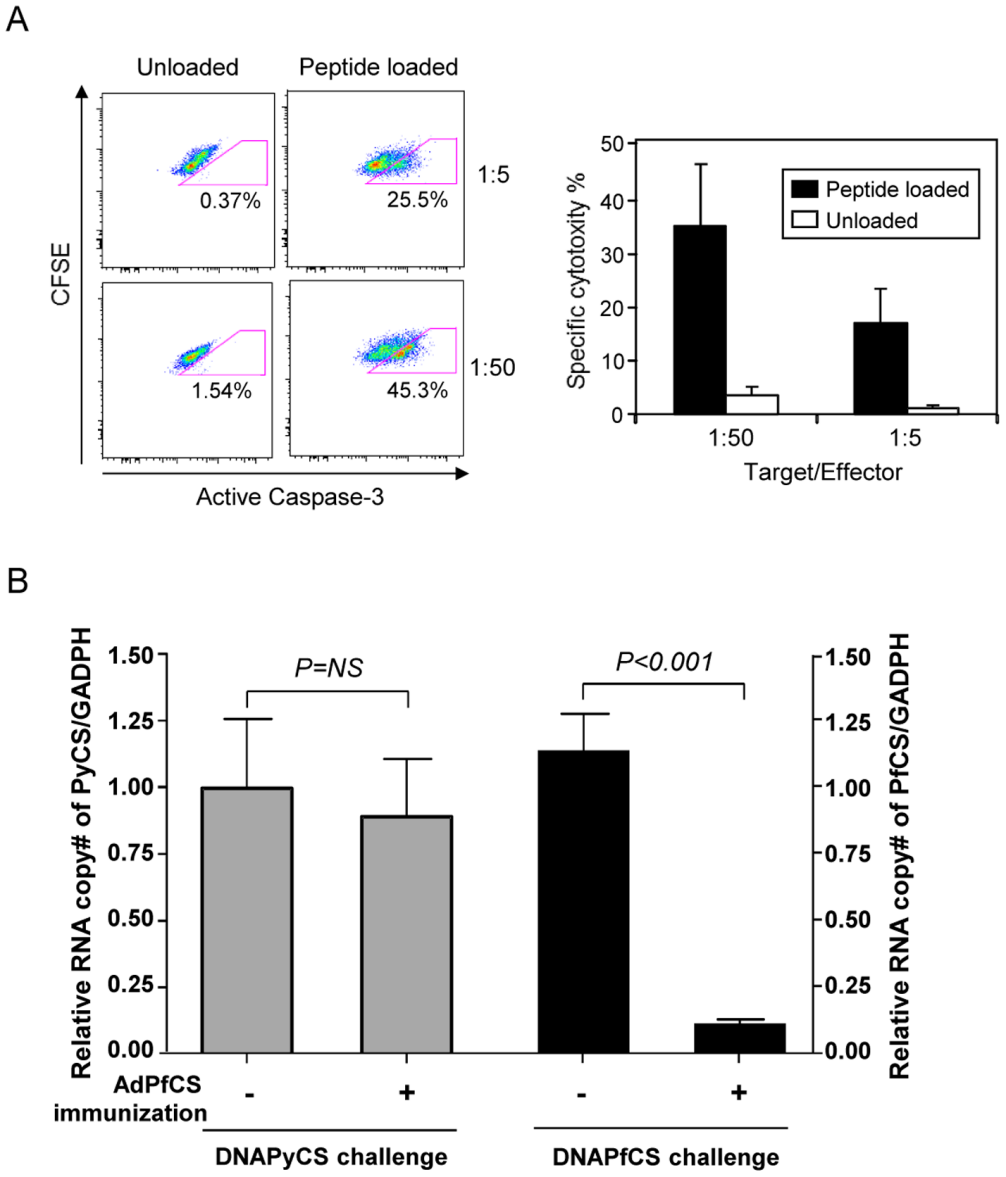
*In vitro* and *in vivo* A2-restricted, PfCS-specific cytotoxic T cell responses induced in AA-A2/hucytokines-transduced HIS mice immunized with AdPfCS. (A) Splenocytes were collected from AAV9-A2/hucytokines transduced HIS mice 2 weeks after AdPfCS immunization. After CD8^+^ T cell enrichment, cells were used as an effector cells. A2^+^ hepatocytes were isolated from AA-A2/hucytokines-transduced NSG mice, labeled with CFSE, and used as target cells. These two cell populations were co-cultured and *in vitro* CTL assays were performed by measuring the amount of caspase 3 within the hepatocytes in the presence or absence of the peptide corresponding to the A2-restricted, CD8^+^ epitope of PfCS antigen. (B) Two weeks after AdPfCS immunization to AAV9-A2/hucytokines-transduced HIS mice, immunized and naïve AAV9-A2/hucytokines-transduced HIS mice were injected with 50 µg of DNA-PfCS dissolved in 2 ml PBS by HTV delivery. Another group of immunized and naïve AAV9-A2/hucytokines-transduced HIS mice received DNA-PyCS via HTV delivery as a negative control. Three days after DNA-PfCS or DNA-PyCS challenge, liver was collected and the amount of PfCS or PyCS mRNA was determined using real-time qRT-PCR.

Finally, to determine the functionality of A2-restricted human CD8^+^ T cells in AAV9-A2/hucytokines-transduced, HSCs-transplanted NSG mice, we determined the cytotoxic effect of the CD8^+^ T cells induced upon vaccination with AdPfCS *in vitro* and *in vivo*. To determine the *in vitro* cytotoxic activity of vaccine-induced, human CD8^+^ T cells in HIS mice, we first isolated hepatocytes from the HIS mice, labeled them with CFSE, and used as target cells. The majority of hepatocytes derived from the HIS mice express both A2 and hβ2m (Figures S1 and S5). Hepatocytes were then co-cultured with CD8^+^ T cells as effector cells (enriched from splenocytes of the HIS mice vaccinated with AdPfCS) in the presence or absence of the peptide corresponding to the A2-restricted CD8^+^ epitope of the PfCS protein. We found that AdPfCS-specific CD8^+^ T cells exert significant cytotoxic activity against peptide-pulsed, but not against non-pulsed, hepatocytes *in vitro*, as determined by caspase-3 activity (Figure 7A). To determine the *in vivo* cytotoxic activity of vaccine-induced, human CD8^+^ T cells in HIS mice, we challenged the mice via hydrodynamic tail vein (HTV) injection with a plasmid DNA encoding either PfCS antigen or PyCS antigen. The HTV injection delivered DNA-PfCSP or DNA-PyCSP to the liver of naïve HIS mice or HIS mice immunized with AdPfCS. Three days after the challenge, expression of each antigen in the liver was determined using real-time qRT-PCR. We found that AdPfCS vaccination to the HIS mice abolished expression of the PfCS antigen, but not of PyCS antigen (Figure 7B). We confirmed that nearly half of the hepatocytes isolated from HIS mice that received HTV injection of DNA-PfCS express HLA-A2 as well PfCS protein using flow cytometric analyses (Figure S5). These results indicate that PfCS antigen-specific, A2-restricted human CD8^+^ T cells induced in AdPfCS-vaccinated HIS mice display cytotoxic activity against A2^+^ hepatocytes expressing the PfCS antigen *in vitro* as well as *in vivo*.

## Discussion

Recent studies have demonstrated that the expression of certain human cytokines dramatically improves reconstitution of specific human-blood lineage cells in humanized mice [5], [10], [11], [23], [56]–[58]. In the current study, we found that among the putative human cytokines known to help hematopoietic cell development, the transduction of a combination of 3 cytokines (human GM-CSF, IL-3, and IL-15 genes) by AAV9 vectors results in the reconstitution of the highest percentage of human CD45^+^ cells (Figure 4). It is important to note that the level of these human cytokines *in vivo* transduced by AAV9 vector-mediated gene delivery became undetectable 16 weeks after AAV9 inoculation, which was 2 weeks before the time of experimental vaccination. In this regard, our mouse model has a great advantage compared to a transgenic mouse model, where the level and duration of cytokine expression cannot be regulated and shut off and, consequently, the continuous presence of human cytokines at the time of vaccination should artificially alter the immunogenicity and efficacy of the vaccine. Another advantage is that the detailed analysis of CMV promoter-driven AAV9 vector-mediated gene expression and localization in various organs of mice after i.v. injection has already been intensively investigated and discussed [36]. In fact, we also performed a comprehensive analysis with regards to the persistence of the AAV9 vector- and transgene-specific GC (Figure 3), and we found that the GC number of the AAV9 vector and the transgenes (hucytokines) became almost undetectable at 20 weeks post injection upon a low dose (1.5**×**10^10^ GC) injection with a cocktail of 3 AAV9 vectors encoding hucytokines (Figure 3). In contrast, the GC number of AAV9 vector and the transgenes (HLA-A2) can remain relatively high for a long period of time when a high dose (1**×**10^11^ GC) of the AAV9-A2 was given, although the GC numbers decreased to some degree in all organs (Figure 3). There are a few possibilities, which may cause the transient expression of human cytokines by AAV9 vectors when being injected at a relatively low dose. Firstly, it is known that AAV enters endosomal compartment upon infecting host cells, presumably resulting in decreased and transient expression of a transgene [59]–[61]. Secondly, one study demonstrated that the integration of the transgene into the infected cell's genome occur only in a small fraction of hepatocytes, thus leading to a transient expression of the transgene in a large fraction of hepatocytes, particularly in young rodents due to rapid turnover of hepatocytes [62]. Finally, although AAV vectors are not immunogenic, host immunity can still react against AAV and eliminate it in a long run [63].

We sought to co-administer NSG mice with AAV9 encoding the 3 human cytokines (AAV9-hucytokines) i.v., together with AAV9 encoding HLA-A2 fused to hβ2m (AAV9-A2) via intrathoracic and i.v. routes. We found that the AAV9-A2/hucytokines-transduced NSG mice were able to reconstitute PBMCs with close to 80% of human CD45^+^ cells, similar to what was observed in A2-Tg NSG mice (Figure 5)[4] and some of BLT mice [18]. Interestingly, these mice could also mount significantly higher numbers of human CD4^+^ and CD8^+^ T cells in their blood than A2-Tg NSG mice (Figure 5). Additionally, we confirmed the expression of both HLA-A2 and hβ2m in the thymic epithelial cells of NSG mice that received intrathoracic injection of AAV9-A2 (Figure 2D and E). Our current studies confirm previous findings that the presence of HLA-class I molecules is required for the development of human CD8^+^ T cells. More importantly, our current findings indicate that the presence of certain human cytokines, particularly at early stages of HSC infusion, not only improves the engraftment and reconstitution of human T cells, but also increases the percentage of effector memory CD8+ T cell subset. The expansion of effector memory CD8+ T cell subset is a trend that we also find in PBMCs collected from a healthy human subject, likely due to the fact that we encounter a myriad of microbial infections during our daily life. Finally, we wish to emphasize that as shown in Figure 5, the variation of the reconstitution status of human immune system, particularly the T cell populations, at 14 weeks post HSC engraftment within a group of NSG mice transduced with AAV9-A2-hucytokines is equivalent to or even less than that observed within a group of A2-Tg NSG mice suggesting that our current AAV vector-mediated gene delivery approach results in a small variation of HIS reconstitution within the group of similarly treated NSG mice. However, the reconstitution status in different experiments is still variable likely due to the derivation of HSCs from different donors. It is also worth mentioning that engrafting a low number of HSCs, i.e. <50,000 per mouse, results in poor HIS reconstitution (data not shown).

With regards to the persistence of the transgene expression by AAV9 vectors, even after 20 weeks post AAV9-A2 inoculation, we could observe a majority of hepatocytes still being able to express HLA-A2 molecules and becoming a target for vaccine-induced, A2-restricted human CD8^+^ T cells, which in turn lyse the hepatocytes both *in vivo* and *in vitro*. The reason for a long duration of A2 expression after AAV9-A2 infection, in contrast to transient expression of AAV9-induced human cytokine expression, is likely due to a high dose, i.e. 1×10^11^ GC, of injection by AAV9-A2, compared to a low dose - 5×10^9^ GC - injection with each AAV9-hucytokine, as demonstrated in Figure 3.

Although human CD8^+^ T cells were present in the peripheral blood of HIS mice, it is important to investigate whether experimental human vaccines can elicit vaccine-specific CD8^+^ T cells in HIS mice. When AAV9-A2/hucytokines-transduced, HSCs-engrafted NSG mice were immunized with adenovirus vaccines expressing malaria and HIV antigens, a high level of vaccine antigen-specific, A2-restricted human CD8^+^ T-cell response was induced in these HIS mice. The CD8^+^ T-cell response was higher than that induced in NSG mice transduced with AAV9-A2 alone or A2-Tg NSG mice that received the same vaccines. Our results agree with previous findings in which the induction of EBV-specific, A2-restricted human CD8^+^ T-cell responses was detected in EBV-infected A2-Tg NSG mice, but not in EBV-infected non-transgenic NSG, or uninfected A2-Tg NSG mice [4], [25]. In addition to EBV, A2-restricted CD8^+^ T cells against other antigens, such as dengue virus-derived antigen [26] and islet antigen [27], have also been shown in A2-Tg NSG mice. In the latter case, the source of human CD34^+^ cells was from a type 1 diabetic patient [27]. HIV-1-specific CD8^+^ T cells restricted by HLA-B51 [64] and HLA-B57 [19] were also shown to be induced in HIV-1-infected B51-Tg NSG mice and BLT mice, respectively.

The most important aspect regarding the functionality of CD8^+^ T cells is their cytotoxic activity. EBV-specific CD8^+^ T cells induced in EBV-infected A2-Tg mice were able to exert A2-restricted cytotoxic activity against EBV-infected human B cells *in vitro* [4]. HIV-specific B57-restricted CD8^+^ T cells were also shown to exert anti-HIV-1 effects in HIV-1-infected BLT mice [19]. Most recently, an islet antigen-specific CD8^+^ T-cell clone was shown to cause beta-cell destruction in A2-Tg NSG mice *in vivo* upon adoptive transfer [65]. In the current study, we are able to demonstrate that malaria antigen-specific A2-restricted human CD8^+^ T cells induced in our HIS mice upon immunization with a human malaria vaccine can exert cytotoxic activity both *in vitro* and *in vivo* in a manner specific to malaria antigen expressed by A2-bearing hepatocytes.

In summary, our study demonstrates that AAV vector-mediated gene delivery is a simple and efficient method that promotes long-term multiple human gene delivery in immune-deficient mice, facilitating the generation of humanized mice and allowing evaluation of the immunogenicity of human vaccines in an efficiently established, small, humanized animal model.

## Supporting Information

Figure S1 **Percentage of HLA-A2^+^/hβ2m^+^ cells in the non-leukocyte (CD45^−^) population residing in the liver, spleen, and bone marrow of AAV9-A2 transduced NSG mice.** Twenty weeks after infection of NSG mice with 5×10^10^ GC of AAV9-A2 by i.v., a single cell suspension was obtained from the liver, spleen, and bone marrow, followed by gating on non-leukocyte (CD45^−^) fraction. The percentages of HLA-A2^+^/hβ2m^+^ cells were determined by flow cytometric analyses.
(TIF)

Figure S2 **Reconstitution of human lymphocytes in the peripheral blood of AAV9-A2/hucytokines transduced, HSCs-engrafted NSG mice.** Flow cytometric analyses were performed to determine the percentage of various human lymphocyte subsets in the blood of various groups of mice 6, 10, and 14 weeks after engraftment of human CD34^+^ cells.
(TIF)

Figure S3 **Reconstitution of human immune system in the spleen and bone marrow of AAV9-A2/hucytokines-transduced, HSCs-engrafted NSG mice.** Flow cytometric analyses were performed to determine the level of various human cell subsets, including human CD45^+^ cells (A), human CD19^+^ B cells (B), and human CD3^+^ T cells (C), in the spleen (Sp) and bone marrow (BM) of various groups of mice 20 weeks after engraftment of HSCs. The groups include; AAV9-A2/hucytokines-transduced NSG mice (N = 5), AAV9-A2-transduced NSG mice (N = 7), A2-Tg NSG mice (N = 5), or AAV9-GFP-transduced NSG mice (N = 4). In (A–C), symbols represent individual percentage and lines represent the mean value for each group. The percentages of human CD8^+^ T cells (D) and CD4^+^ T cells (E) within the human CD3^+^ T cells in spleen are also shown in symbols and lines for individual percentage and the mean value, respectively. The mean absolute numbers of CD8^+^ and CD4^+^ T cells in 5×10^5^ splenocytes are shown in the gray bar graphs with standard errors. The statistical differences refer to the difference among the percentages in (A–C) and the absolute numbers in (D) and (E). *p<0.05; **p<0.01; ***p<0.001.
(TIF)

Figure S4 **Reconstitution of human CD34^+^HLA-A2^+^ in the bone marrow of AAV9-A2/hucytokines-transduced, HSCs-engrafted NSG mice.** Flow cytometric analyses were performed to determine the level of human CD34^+^HLA-A2^+^ (HSC lineage markers) in total bone marrow cells of various groups of NSG mice 20 weeks after engraftment of HSCs. The groups include; AAV9-A2/hucytokines-transduced NSG mice (N = 5), AAV9-A2-transduced NSG mice (N = 7), A2-Tg NSG mice (N = 5), or AAV9-GFP-transduced NSG mice (N = 4). ***p<0.001.
(TIF)

Figure S5 **Co-expression of HLA-A2 and PfCS antigen in hepatocytes isolated from AAV9-A2-transduced NSG mice challenged with DNA-PfCS by HTV delivery.** Sixteen weeks after infecting NSG mice with AAV9-A2, 50 µg of a plasmid encoding PfCS dissolved in 2 ml PBS was injected in the mice by HTV delivery. After 3 days, hepatocytes were isolated by liver perfusion, and co-expression of HLA-A2 and PfCS antigen was determined by flow cytometric analyses.
(TIF)

## References

[1] Bosma GC, Custer RP, Bosma MJ (1983) A severe combined immunodeficiency mutation in the mouse. Nature 301: 527–530. 6823332 10.1038/301527a0

[2] Mosier DE, Gulizia RJ, Baird SM, Wilson DB (1988) Transfer of a functional human immune system to mice with severe combined immunodeficiency. Nature 335: 256–259. 2970594 10.1038/335256a0

[3] Ishikawa F, Yasukawa M, Lyons B, Yoshida S, Miyamoto T, et al (2005) Development of functional human blood and immune systems in NOD/SCID/IL2 receptor {gamma} chain(null) mice. Blood 106: 1565–1573. 15920010 10.1182/blood-2005-02-0516 PMC1895228

[4] Shultz LD, Saito Y, Najima Y, Tanaka S, Ochi T, et al (2010) Generation of functional human T-cell subsets with HLA-restricted immune responses in HLA class I expressing NOD/SCID/IL2r gamma(null) humanized mice. Proc Natl Acad Sci USA 107: 13022–13027. 20615947 10.1073/pnas.1000475107 PMC2919921

[5] Tanaka S, Saito Y, Kunisawa J, Kurashima Y, Wake T, et al (2012) Development of mature and functional human myeloid subsets in hematopoietic stem cell-engrafted NOD/SCID/IL2rγKO mice. J Immunol 188: 6145–6155. 22611244 10.4049/jimmunol.1103660 PMC3370073

[6] Lepus CM, Gibson TF, Gerber SA, Kawikova I, Szczepanik M, et al (2009) Comparison of human fetal liver, umbilical cord blood, and adult blood hematopoietic stem cell engraftment in NOD-scid/gammac-/-, Balb/c-Rag1-/-gammac-/-, and C.B-17-scid/bg immunodeficient mice. Hum Immunol 70: 790–802. 19524633 10.1016/j.humimm.2009.06.005 PMC2949440

[7] Shultz LD, Lyons BL, Burzenski LM, Gott B, Chen X, et al (2005) Human lymphoid and myeloid cell development in NOD/LtSz-scid IL2R gamma null mice engrafted with mobilized human hematopoietic stem cells. J Immunol 174: 6477–6489. 15879151 10.4049/jimmunol.174.10.6477

[8] Matsumura T, Kametani Y, Ando K, Hirano Y, Katano I, et al (2003) Functional CD5+ B cells develop predominantly in the spleen of NOD/SCID/gammac(null) (NOG) mice transplanted either with human umbilical cord blood, bone marrow, or mobilized peripheral blood CD34+ cells. Exp Hematol 31: 789–797. 12962725 10.1016/s0301-472x(03)00193-0

[9] Watanabe Y, Takahashi T, Okajima A, Shiokawa M, Ishii N, et al (2009) The analysis of the functions of human B and T cells in humanized NOD/shi-scid/gammac(null) (NOG) mice (hu-HSC NOG mice). Int Immunol 21: 843–858. 19515798 10.1093/intimm/dxp050

[10] Chen Q, Khoury M, Chen J (2009) Expression of human cytokines dramatically improves reconstitution of specific human-blood lineage cells in humanized mice. Proc Natl Acad Sci U S A 106: 21783–21788. 19966223 10.1073/pnas.0912274106 PMC2789167

[11] Billerbeck E, Barry WT, Mu K, Dorner M, Rice CM, et al (2011) Development of humanmCD4+FoxP3+ regulatory T cells in human stem cell factor-, granulocyte-macrophage mcolony-stimulating factor-, and interleukin-3-expressing NOD-SCID IL2Rγ(null) humanized mice. Blood 117: 3076–3086. 21252091 10.1182/blood-2010-08-301507 PMC3062310

[12] Wege AK, Melkus MW, Denton PW, Estes JD, Garcia JV (2008) Functional and phenotypic characterization of the humanized BLT mouse model. Curr Top Microbiol Immunol 324: 149–165. 18481459 10.1007/978-3-540-75647-7_10

[13] Lan P, Tonomura N, Shimizu A, Wang S, Yang YG (2006) Reconstitution of a functional human immune system in immunodeficient mice through combined human fetal thymus/liver and CD34+ cell transplantation. Blood 108: 487–492. 16410443 10.1182/blood-2005-11-4388

[14] Melkus MW, Estes JD, Padgett-Thomas A, Gatlin J, Denton PW, et al (2006) Humanized mice mount specific adaptive and innate immune responses to EBV and TSST-1. Nat Med 12: 1316–1322. 17057712 10.1038/nm1431

[15] Brainard DM, Seung E, Frahm N, Cariappa A, Bailey CC, et al (2009) Induction of robust cellular and humoral virus-specific adaptive immune responses in human immunodeficiency virus-infected humanized BLT mice. J Virol 83: 7305–7321. 19420076 10.1128/JVI.02207-08 PMC2704767

[16] Marsden MD, Kovochich M, Suree N, Shimizu S, Mehta R, et al (2011) HIV latency in the humanized BLT mouse. J Virol 86: 339–347. 22072769 10.1128/JVI.06366-11 PMC3255908

[17] Denton PW, Olesen R, Choudhary SK, Archin NM, Wahl A, et al (2012) Generation of HIV latency in humanized BLT mice. J Virol 86: 630–634. 22013053 10.1128/JVI.06120-11 PMC3255928

[18] Greenblatt MB, Vrbanac V, Tivey T, Tsang K, Tager AM, et al (2012) Graft versus Host Disease in the Bone Marrow, Liver and Thymus Humanized Mouse Model. PLoS One 7: e44664. 22957096 10.1371/journal.pone.0044664 PMC3434179

[19] Dudek TE, No DC, Seung E, Vrbanac VD, Fadda L, et al (2012) Rapid evolution of HIV-1 to functional CD8^+^ T cell responses in humanized BLT mice. Sci Transl Med 4: 143ra98. 10.1126/scitranslmed.3003984 PMC3685142 22814851

[20] Wahl A, Swanson MD, Nochi T, Olesen R, Denton PW, et al (2012) Human breast milk and antiretrovirals dramatically reduce oral HIV-1 transmission in BLT humanized mice. PLoS Pathog 8: e1002732. 22737068 10.1371/journal.ppat.1002732 PMC3380612

[21] Zou W, Denton PW, Watkins RL, Krisko JF, Nochi T, et al (2012) Nef functions in BLT mice to enhance HIV-1 replication and deplete CD4+CD8+ thymocytes. Retrovirology 9: 44. 22640559 10.1186/1742-4690-9-44 PMC3403983

[22] Jaiswal S, Pazoles P, Woda M, Shultz LD, Greiner DL, et al (2012) Enhanced humoral and HLA-A2-restricted dengue virus-specific T-cell responses in humanized BLT NSG mice. Immunology 136: 334–343. 22384859 10.1111/j.1365-2567.2012.03585.x PMC3385033

[23] Willinger T, Rongvaux A, Takizawa H, Yancopoulos GD, Valenzuela DM, et al (2011) Human IL-3/GM-CSF knock-in mice support human alveolar macrophage development and human immune responses in the lung. Proc Natl Acad Sci U S A 108: 2390–2395. 21262803 10.1073/pnas.1019682108 PMC3038773

[24] Strowig T, Rongvaux A, Rathinam C, Takizawa H, Borsotti C, et al (2011) Transgenic expression of human signal regulatory protein alpha in Rag2-/-gamma(c)-/- mice improves engraftment of human hematopoietic cells in humanized mice. Proc Natl Acad Sci U S A 108: 13218–13223. 21788509 10.1073/pnas.1109769108 PMC3156175

[25] Strowig T, Gurer C, Ploss A, Liu YF, Arrey F, et al (2009) Priming of protective T cell responses against virus-induced tumors in mice with human immune system components. J Exp Med 206: 1423–1434. 19487422 10.1084/jem.20081720 PMC2715061

[26] Jaiswal S, Pearson T, Friberg H, Shultz LD, Greiner DL, et al (2009) Dengue virus infection and virus-specific HLA-A2 restricted immune responses in humanized NOD-scid IL2rgammanull mice. PLoS One 4: e7251. 19802382 10.1371/journal.pone.0007251 PMC2749937

[27] Whitfield-Larry F, Young EF, Talmage G, Fudge E, Azam A, et al (2011) HLA-A2-matched peripheral blood mononuclear cells from type 1 diabetic patients, but not nondiabetic donors, transfer insulitis to NOD-scid/γc(null)/HLA-A2 transgenic mice concurrent with the expansion of islet-specific CD8+ T cells. Diabetes 60: 1726–1733. 21521873 10.2337/db10-1287 PMC3114397

[28] Danner R, Chaudhari SN, Rosenberger J, Surls J, Richie TL, et al (2011) Expression of HLA class II molecules in humanized NOD.Rag1KO.IL2RgcKO mice is critical for development and function of human T and B cells. PLoS One 6: e19826. 21611197 10.1371/journal.pone.0019826 PMC3096643

[29] Suzuki M, Takahashi T, Katano I, Ito R, Ito M, et al (2012) Induction of human humoral immune responses in a novel HLA-DR-expressing transgenic NOD/Shi-scid/γcnull mouse. Int Immunol 24: 243–252. 22402880 10.1093/intimm/dxs045

[30] Flotte TR, Carter BJ (1995) Adeno-associated virus vectors for gene therapy. Gene Ther 2: 357–362. 7584109

[31] Hicks MJ, Rosenberg JB, De BP, Pagovich OE, Young CN, et al (2012) AAV-directed persistent expression of a gene encoding anti-nicotine antibody for smoking cessation. Sci Transl Med 4: 140ra87. 10.1126/scitranslmed.3003611 PMC3622954 22745437

[32] Balazs AB, Chen J, Hong CM, Rao DS, Yang L, et al (2011) Antibody-based protection against HIV infection by vectored immunoprophylaxis. Nature 481: 81–84. 22139420 10.1038/nature10660 PMC3253190

[33] Watanabe M, Boyer JL, Crystal RG (2010) AAVrh.10-mediated genetic delivery of bevacizumab to the pleura to provide local anti-VEGF to suppress growth of metastatic lung tumors. Gene Ther 17: 1042–1051. 20596059 10.1038/gt.2010.87 PMC2921016

[34] Skaricic D, Traube C, De B, Joh J, Boyer J, et al (2008) Genetic delivery of an anti-RSV antibody to protect against pulmonary infection with RSV. Virology 378: 79–85. 18556039 10.1016/j.virol.2008.04.016

[35] Dion S, Bourgine M, Godon O, Levillayer F, Michel ML (2013) Adeno-associated virus-mediated gene transfer leads to persistent hepatitis B virus replication in mice expressing HLA-A2 and HLA-DR1 molecules. J Virol 87: 5554–5563. 23468504 10.1128/JVI.03134-12 PMC3648192

[36] Zincarelli C, Soltys S, Rengo G, Rabinowitz JE (2008) Analysis of AAV serotypes 1-9 mediated gene expression and tropism in mice after systemic injection. Mol Ther 16: 1073–1080. 18414476 10.1038/mt.2008.76

[37] Kitchen SG, Levin BR, Bristol G, Rezek V, Kim S, et al (2012) In Vivo Suppression of HIV by Antigen Specific T Cells Derived from Engineered Hematopoietic Stem Cells. PLoS Pathog 8: e1002649. 22511873 10.1371/journal.ppat.1002649 PMC3325196

[38] Vitiello A, Marchesini D, Furze J, Sherman LA, Chesnut RW (1991) Analysis of the HLA-restricted influenza-specific cytotoxic T lymphocyte response in transgenic mice carrying a chimeric human-mouse class I major histocompatibility complex. J Exp Med 173: 1007–1015. 1706750 10.1084/jem.173.4.1007 PMC2190816

[39] Marks-Konczalik J, Dubois S, Losi JM, Sabzevari H, Yamada N, et al (2000) IL-2-induced activation-induced cell death is inhibited in IL-15 transgenic mice. Proc Natl Acad Sci U S A 97: 11445–11450. 11016962 10.1073/pnas.200363097 PMC17219

[40] Lin J, Zhi Y, Mays L, Wilson JM (2007) Vaccines based on novel adeno-associated virus vectors elicit aberrant CD8+ T-cell responses in mice. J Virol 81: 11840–11849. 17715240 10.1128/JVI.01253-07 PMC2168776

[41] Wang L, Calcedo R, Nichols TC, Bellinger DA, Dillow A, et al (2005) Sustained correction of disease in naive and AAV2-pretreated hemophilia B dogs: AAV2/8-mediated, liver-directed gene therapy. Blood 105: 3079–3086. 15637142 10.1182/blood-2004-10-3867

[42] Ghosh A, Yue Y, Duan D (2006) Viral serotype and the transgene sequence influence overlapping adeno-associated viral (AAV) vector-mediated gene transfer in skeletal muscle. J Gene Med 8: 298–305. 16385549 10.1002/jgm.835 PMC2581716

[43] Paul D, Qazilbash MH, Song K, Xu H, Sinha BK, et al (2000) Construction of a recombinant adeno-associated virus (rAAV) vector expressing murine interleukin-12 (IL-12). Cancer Gene Ther 7: 308–315. 10770641 10.1038/sj.cgt.7700105

[44] Wendtner CM, Kofler DM, Theiss HD, Kurzeder C, Buhmann R, et al (2002) Efficient gene transfer of CD40 ligand into primary B-CLL cells using recombinant adeno-associated virus (rAAV) vectors. Blood 100: 1655–1661. 12176885

[45] Rai U, Huang J, Mishra S, Li X, Shiratsuchi T, Tsuji M (2012) A new method to determine antigen-specific CD8+ T cell activity in vivo by hydrodynamic injection. Biomolecules 2: 23–33. 24970125 10.3390/biom2010023 PMC4030865

[46] Nelson AJ, Dunn RJ, Peach R, Aruffo A, Farr AG (1996) The murine homolog of human Ep-CAM, a homotypic adhesion molecule, is expressed by thymocytes and thymic epithelial cells. Eur J Immunol 26: 401–408. 8617310 10.1002/eji.1830260220

[47] Shiratsuchi T, Rai U, Krause A, Worgall S, Tsuji M (2010) Replacing adenoviral vector HVR1 with a malaria B cell epitope improves immunogenicity and circumvents preexisting immunity to adenovirus in mice. J Clin Invest 120: 3688–36701. 20811151 10.1172/JCI39812 PMC2947213

[48] Li X, Fujio M, Imamura M, Wu D, Vasan S, et al (2010) Design of a potent CD1d-binding NKT cell ligand as a vaccine adjuvant. Proc Natl Acad Sci U S A 107: 13010–13015. 20616071 10.1073/pnas.1006662107 PMC2919905

[49] Blum-Tirouvanziam U, Servis C, Habluetzel A, Valmori D, Men Y, et al (1995) Localization of HLA-A2.1-restricted T cell epitopes in the circumsporozoite protein of Plasmodium falciparum. J Immunol 154: 3922–3931. 7535817

[50] Nixon DF, Townsend AR, Elvin JG, Rizza CR, Gallwey J, et al (1988) HIV-1 gag-specific cytotoxic T lymphocytes defined with recombinant vaccinia virus and synthetic peptides. Nature 336: 484–487. 2461519 10.1038/336484a0

[51] Li WC, Ralphs KL, Tosh D (2010) Isolation and culture of adult mouse hepatocytes. Methods Mol Biol 633: 185–196. 20204628 10.1007/978-1-59745-019-5_13

[52] Fujioka H, Hunt PJ, Rozga J, Wu GD, Cramer DV, et al (1994) Carboxyfluorescein (CFSE) labelling of hepatocytes for short-term localization following intraportal transplantation. Cell Transplant 3: 397–408. 7827777 10.1177/096368979400300506

[53] Jerome KR, Sloan DD, Aubert M (2003) Measurement of CTL-induced cytotoxicity: The caspase 3 assay. Apoptosis 8: 563–571. 14574062 10.1023/A:1026123223387

[54] He L, Hakimi J, Salha D, Miron I, Dunn P, et al (2005) A sensitive flow cytometry-based cytotoxic T-lymphocyte assay through detection of cleaved caspase 3 in target cells. J Immunol Methods 304: 43–59. 16076473 10.1016/j.jim.2005.06.005

[55] Zhang G, Budker V, Wolff JA (1999) High levels of foreign gene expression in hepatocytes after tail vein injections of naked plasmid DNA. Hum. Gene Ther 10: 1735–1737. 10.1089/10430349950017734 10428218

[56] Takagi S, Saito Y, Hijikata A, Tanaka S, Watanabe T, et al (2012) Membrane-bound human SCF/KL promotes in vivo human hematopoietic engraftment and myeloid differentiation. Blood 119: 2768–2777. 22279057 10.1182/blood-2011-05-353201 PMC3327455

[57] Brehm MA, Racki WJ, Leif J, Burzenski L, Hosur V, et al (2012) Engraftment of human HSCs in nonirradiated newborn NOD-scid IL2rγ null mice is enhanced by transgenic expression of membrane-bound human SCF. Blood 119: 2778–2788. 22246028 10.1182/blood-2011-05-353243 PMC3327456

[58] Cimbro R, Vassena L, Arthos J, Cicala C, Kehrl JH, et al (2012) IL-7 induces expression and activation of integrin α4β7 promoting naive T-cell homing to the intestinal mucosa. Blood 120: 2610–2619. 22896005 10.1182/blood-2012-06-434779 PMC3460683

[59] Hansen J, Qing K, Srivastava A (2001) Adeno-associated virus type 2-mediated gene transfer: altered endocytic processing enhances transduction efficiency in murine fibroblasts. J Virol 75: 4080–4090. 11287557 10.1128/JVI.75.9.4080-4090.2001 PMC114153

[60] Douar AM, Poulard K, Stockholm D, Danos O (2001) Intracellular trafficking of adeno-associated virus vectors: routing to the late endosomal compartment and proteasome degradation. J Virol 75: 1824–1833. 11160681 10.1128/JVI.75.4.1824-1833.2001 PMC114092

[61] Ding W, Zhang L, Yan Z, Engelhardt JF (2005) Intracellular trafficking of adeno-associated viral vectors. Gene Ther 12: 873–880. 15829993 10.1038/sj.gt.3302527

[62] Flageul M, Aubert D, Pichard V, Nguyen TH, Nowrouzi A, et al (2009) Transient expression of genes delivered to newborn rat liver using recombinant adeno-associated virus 2/8 vectors. J. Gene Med 11: 689–696. 19455564 10.1002/jgm.1343

[63] Mingozzi F, High KA (2011) Immune responses to AAV in clinical trials. Curr Gene Ther 11: 321–330. 21557723 10.2174/156652311796150354

[64] Sato Y, Nagata S, Takiguchi M (2012) Effective elicitation of human effector CD8+ T Cells in HLA-B*51:01 transgenic humanized mice after infection with HIV-1. PLoS One 7: e42776. 22880104 10.1371/journal.pone.0042776 PMC3412802

[65] Unger WW, Pearson T, Abreu JR, Laban S, van der Slik AR, et al (2012) Islet-specific CTL cloned from a type 1 diabetes patient cause beta-cell destruction after engraftment into HLA-A2 transgenic NOD/scid/IL2RG null mice. PLoS One 7: e49213. 23155466 10.1371/journal.pone.0049213 PMC3498321

