# Supplementary figures and images for: An AAV Vector-Mediated Gene Delivery Approach Facilitates Reconstitution of Functional Human CD8+ T Cells in Mice
Source: PLoS One. 2014 Feb 6;9(2):e88205. doi: 10.1371/journal.pone.0088205 (PMC3916402; doi:10.1371/journal.pone.0088205)

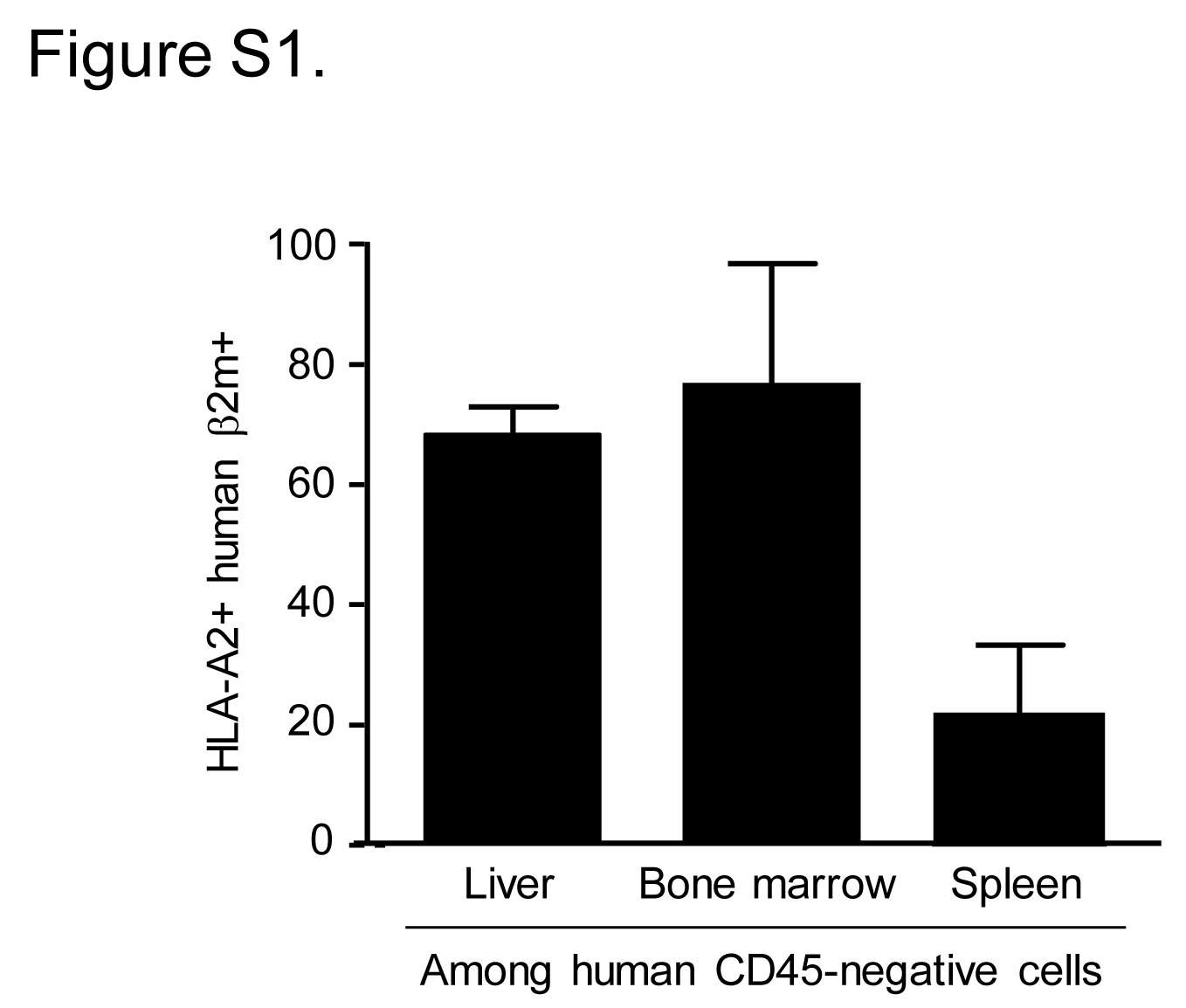

Supplement: Figure S1 — Percentage of HLA-A2+/hβ2m+ cells in the non-leukocyte (CD45−) population residing in the liver, spleen, and bone marrow of AAV9-A2 transduced NSG mice. Twenty weeks after infection of NSG mice with 5×10^10^ GC of AAV9-A2 by i.v., a single cell suspension was obtained from the liver, spleen, and bone marrow, followed by gating on non-leukocyte (CD45−) fraction. The percentages of HLA-A2+/hβ2m+ cells were determined by flow cytometric analyses. (TIF) [file pone.0088205.s001.tif]

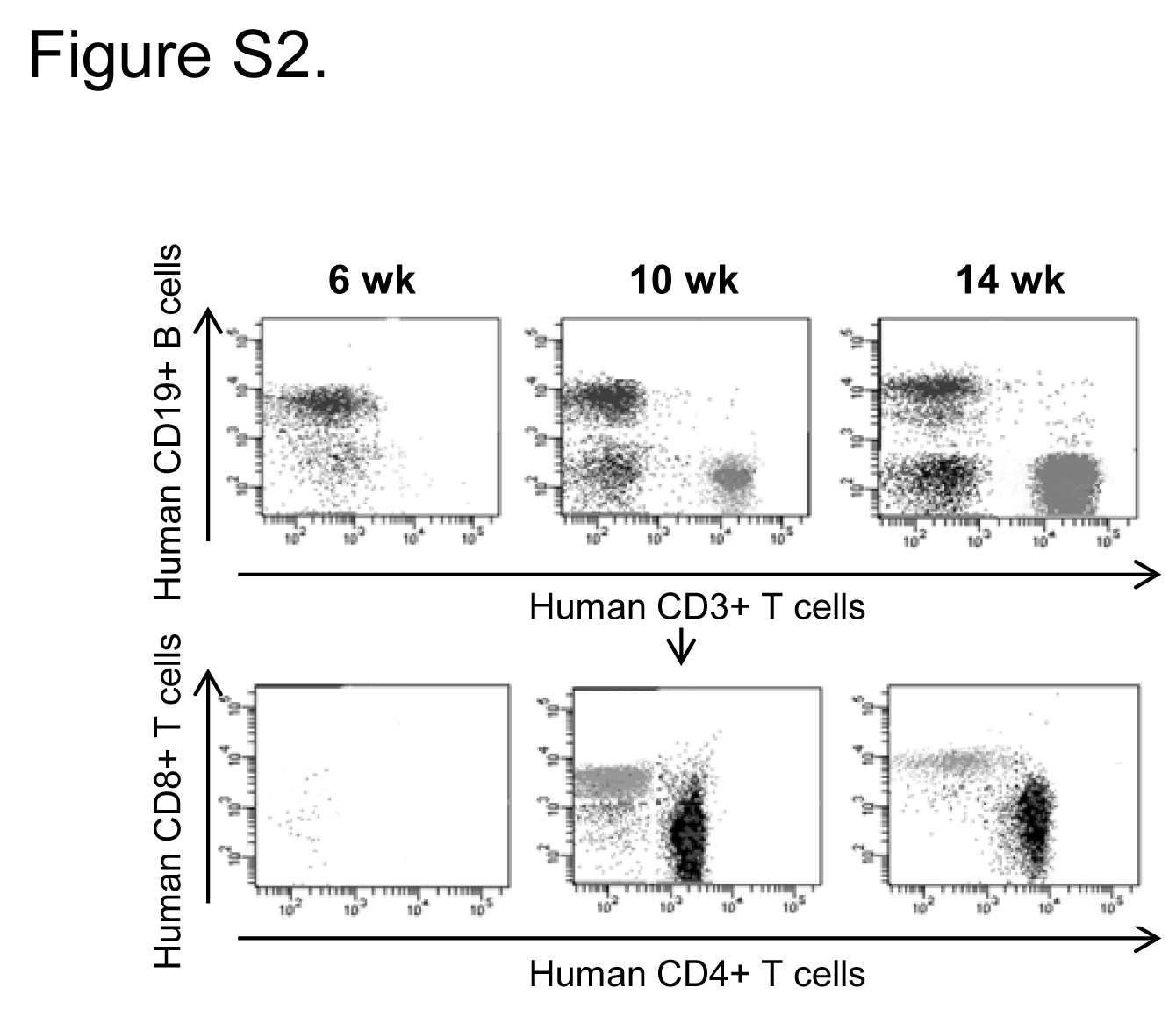

Supplement: Figure S2 — Reconstitution of human lymphocytes in the peripheral blood of AAV9-A2/hucytokines transduced, HSCs-engrafted NSG mice. Flow cytometric analyses were performed to determine the percentage of various human lymphocyte subsets in the blood of various groups of mice 6, 10, and 14 weeks after engraftment of human CD34+ cells. (TIF) [file pone.0088205.s002.tif]

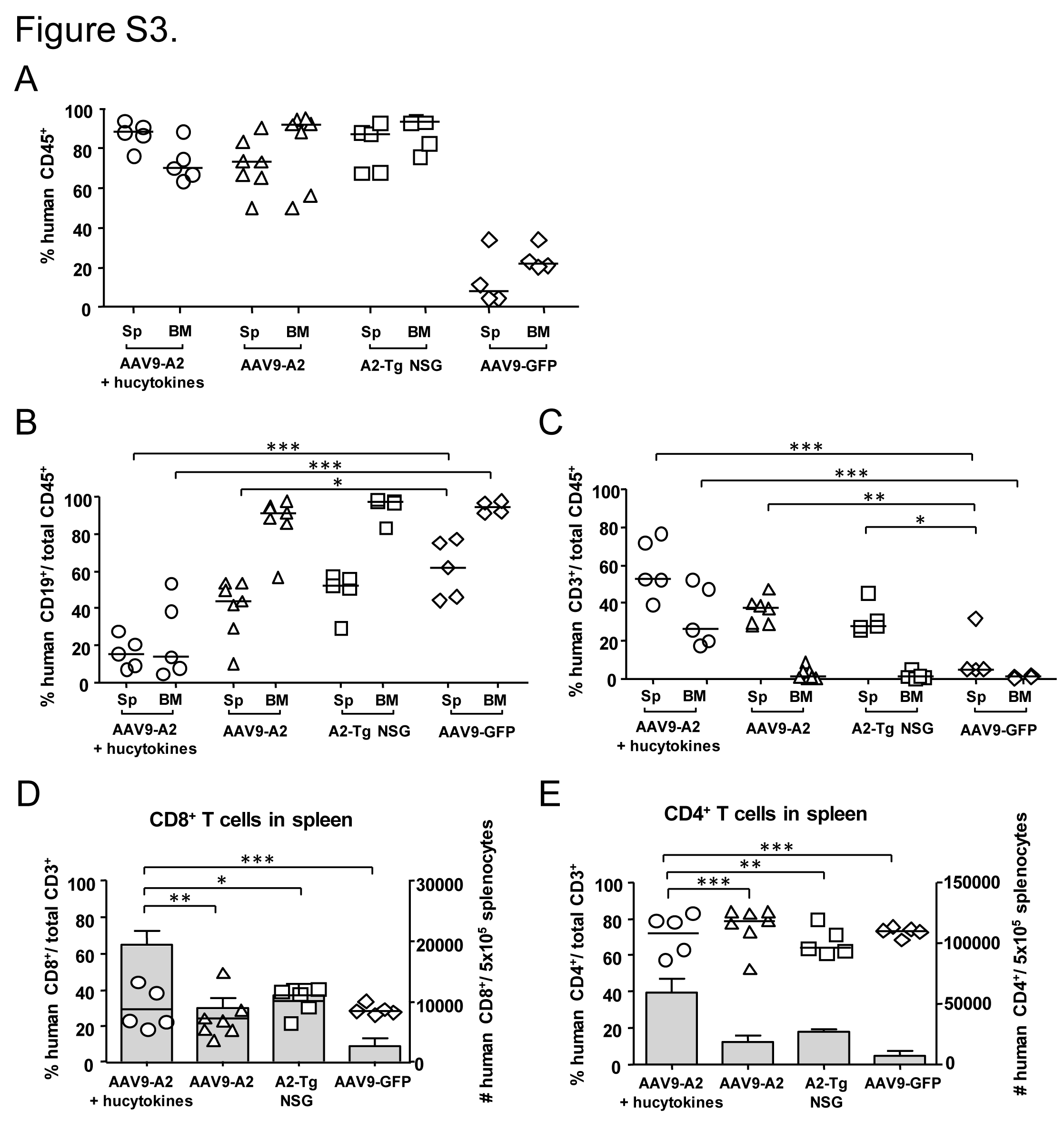

Supplement: Figure S3 — Reconstitution of human immune system in the spleen and bone marrow of AAV9-A2/hucytokines-transduced, HSCs-engrafted NSG mice. Flow cytometric analyses were performed to determine the level of various human cell subsets, including human CD45+ cells (A), human CD19+ B cells (B), and human CD3+ T cells (C), in the spleen (Sp) and bone marrow (BM) of various groups of mice 20 weeks after engraftment of HSCs. The groups include; AAV9-A2/hucytokines-transduced NSG mice (N = 5), AAV9-A2-transduced NSG mice (N = 7), A2-Tg NSG mice (N = 5), or AAV9-GFP-transduced NSG mice (N = 4). In (A–C), symbols represent individual percentage and lines represent the mean value for each group. The percentages of human CD8+ T cells (D) and CD4+ T cells (E) within the human CD3+ T cells in spleen are also shown in symbols and lines for individual percentage and the mean value, respectively. The mean absolute numbers of CD8+ and CD4+ T cells in 5×10^5^ splenocytes are shown in the gray bar graphs with standard errors. The statistical differences refer to the difference among the percentages in (A–C) and the absolute numbers in (D) and (E). *p<0.05; **p<0.01; ***p<0.001. (TIF) [file pone.0088205.s003.tif]

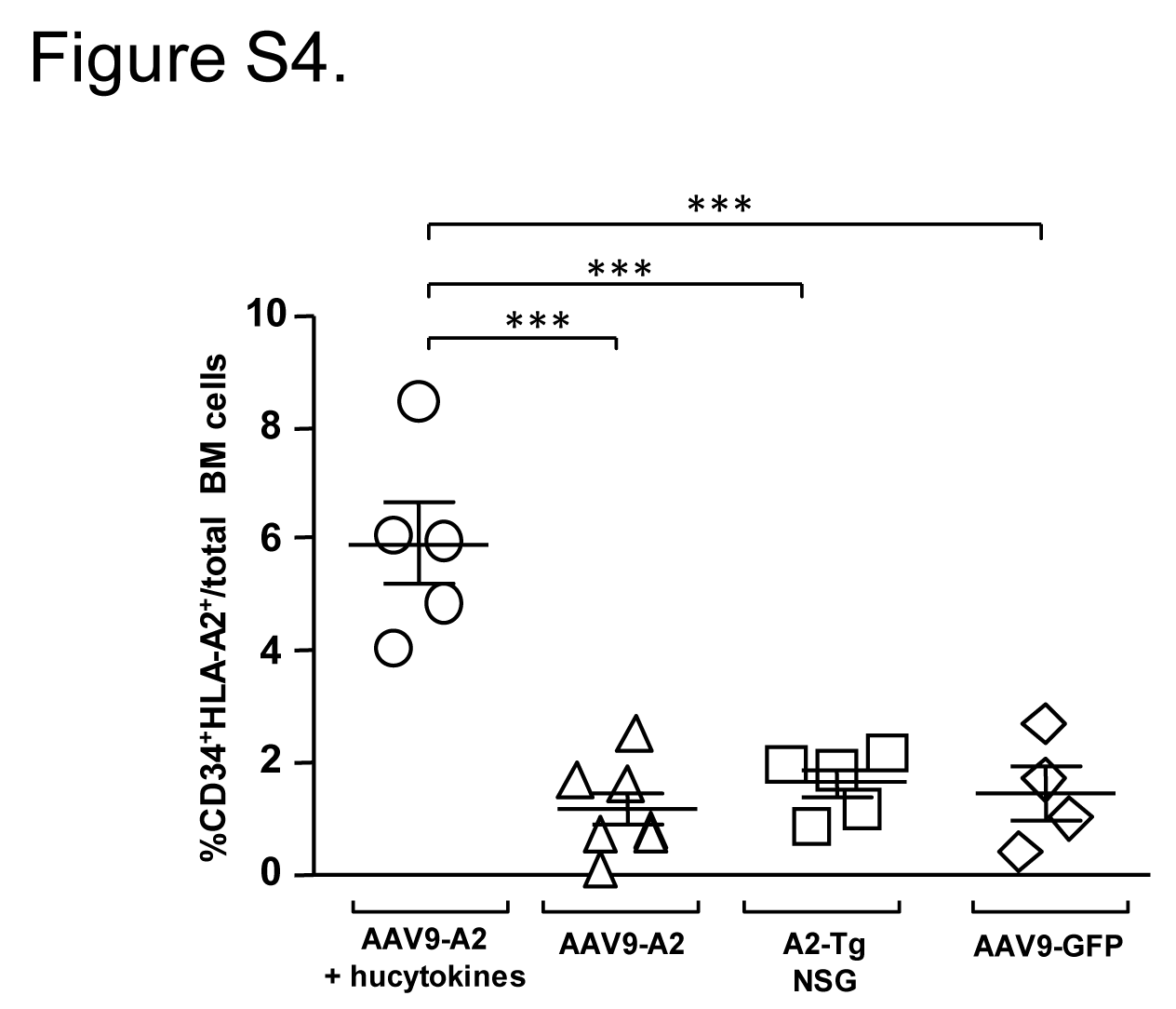

Supplement: Figure S4 — Reconstitution of human CD34+HLA-A2+ in the bone marrow of AAV9-A2/hucytokines-transduced, HSCs-engrafted NSG mice. Flow cytometric analyses were performed to determine the level of human CD34+HLA-A2+ (HSC lineage markers) in total bone marrow cells of various groups of NSG mice 20 weeks after engraftment of HSCs. The groups include; AAV9-A2/hucytokines-transduced NSG mice (N = 5), AAV9-A2-transduced NSG mice (N = 7), A2-Tg NSG mice (N = 5), or AAV9-GFP-transduced NSG mice (N = 4). ***p<0.001. (TIF) [file pone.0088205.s004.tif]

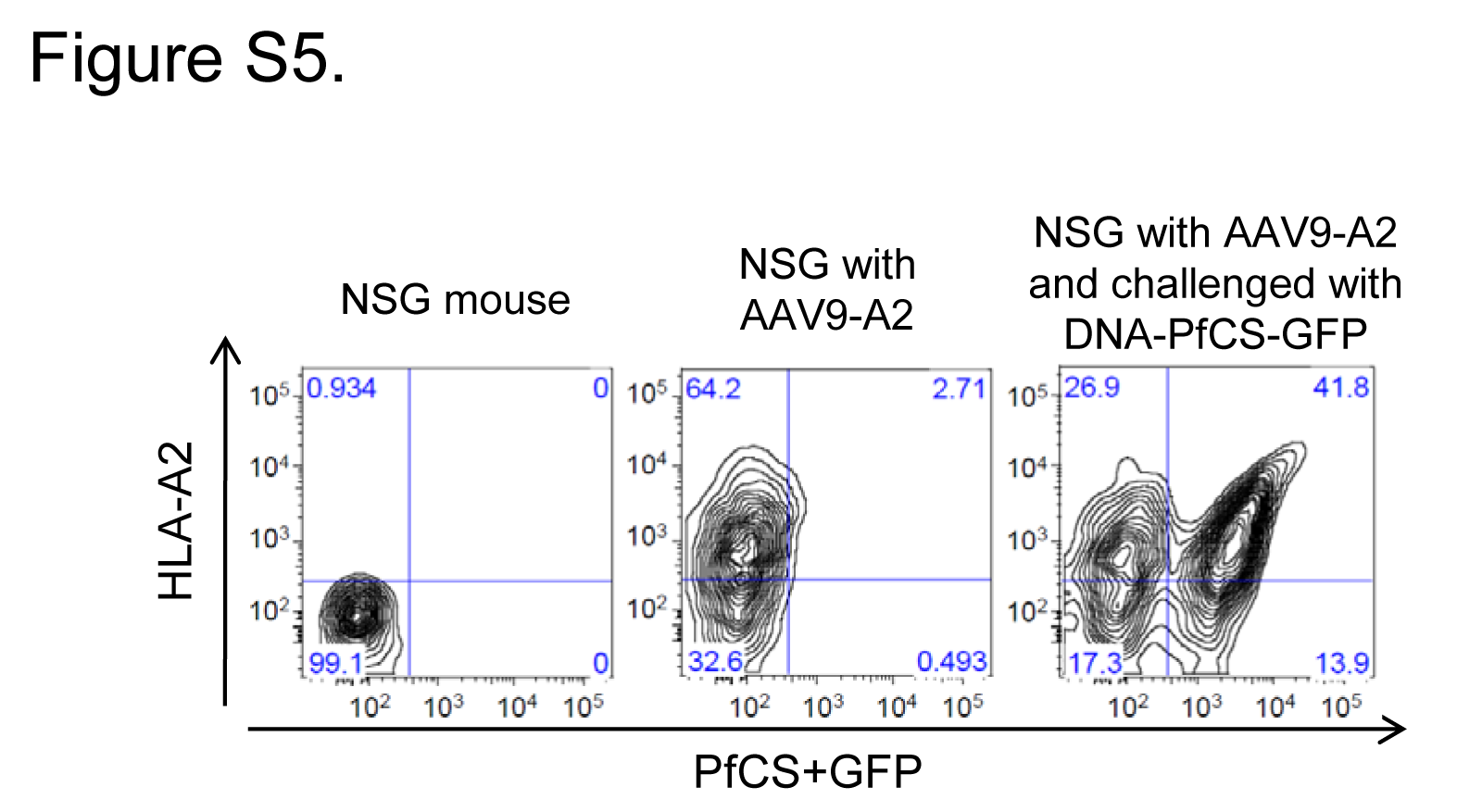

Supplement: Figure S5 — Co-expression of HLA-A2 and PfCS antigen in hepatocytes isolated from AAV9-A2-transduced NSG mice challenged with DNA-PfCS by HTV delivery. Sixteen weeks after infecting NSG mice with AAV9-A2, 50 µg of a plasmid encoding PfCS dissolved in 2 ml PBS was injected in the mice by HTV delivery. After 3 days, hepatocytes were isolated by liver perfusion, and co-expression of HLA-A2 and PfCS antigen was determined by flow cytometric analyses. (TIF) [file pone.0088205.s005.tif]
